# *DNMT2*/*TRDMT1* gene knockout compromises doxorubicin-induced unfolded protein response and sensitizes cancer cells to ER stress-induced apoptosis

**DOI:** 10.1007/s10495-022-01779-0

**Published:** 2022-10-23

**Authors:** Jagoda Adamczyk-Grochala, Dominika Bloniarz, Klaudia Zielinska, Anna Lewinska, Maciej Wnuk

**Affiliations:** Department of Biotechnology, Institute of Biology and Biotechnology, College of Natural Sciences, University of Rzeszow, Pigonia 1, 35-310 Rzeszow, Poland

**Keywords:** DNMT2/TRDMT1, Cancer cells, ER stress, Doxorubicin, MicroRNA expression

## Abstract

The acidic, hypoxic and nutrient-deprived tumor microenvironment may induce endoplasmic reticulum (ER) stress and the unfolded protein response (UPR) may exert an important cytoprotective role by promoting folding of newly synthesized proteins and cancer cell survival. The lack of DNMT2/TRDMT1 methyltransferase-mediated C38 tRNA methylation compromises translational fidelity that may result in the accumulation of misfolded and aggregated proteins leading to proteotoxic stress-related cell death. In the present study, *DNMT2/TRDMT1* gene knockout-mediated effects were investigated during doxorubicin (DOX)-induced ER stress and PERK-, IRE1- and ATF6-orchestrated UPR in four genetically different cellular models of cancer (breast and cervical cancer, osteosarcoma and glioblastoma cells). Upon DOX stimulation, *DNMT2/TRDMT1* gene knockout impaired PERK activation and modulated NSUN and 5-methylcytosine RNA-based responses and microRNA profiles. The lack of *DNMT2/TRDMT1* gene in DOX-treated four cancer cell lines resulted in decreased levels of four microRNAs, namely, miR-23a-3p, miR-93-5p, miR-125a-5p and miR-191-5p involved in the regulation of several pathways such as ubiquitin-mediated proteolysis, amino acid degradation and translational misregulation in cancer. We conclude that *DNMT2/TRDMT1* gene knockout, at least in selected cellular cancer models, affects adaptive responses associated with protein homeostasis networks that during prolonged ER stress may result in increased sensitivity to apoptotic cell death.

**Supplementary Information:**

The online version contains supplementary material available at 10.1007/s10495-022-01779-0.

## Introduction

The endoplasmic reticulum (ER), a multifunctional organelle, is the first compartment of the secretory pathway involved in protein folding and quality control [1]. ER-mediated proteostasis may be affected by a plethora of endogenous and exogenous stimuli/stressors [1]. The accumulation of unfolded or misfolded proteins in the ER lumen, a sign of proteotoxicity and ER stress, results in the induction of the unfolded protein response (UPR), an adaptive signaling network that is essential for cell survival [2–4]. The UPR is initiated by the activation of three ER membrane proteins and stress sensors, namely inositol-requiring enzyme 1 (IRE1), protein kinase RNA-activated (PKR)-like ER kinase (PERK) and activating transcription factor 6 (ATF6) [2, 3]. The action of IRE1, PERK and ATF6 stress transducers and their downstream effectors decreases ER protein load, promotes ER protein folding and stimulates elimination of misfolded proteins that restores ER proteostasis [2, 3]. However, if severe ER stress is induced or the UPR is prolonged, ER homeostasis cannot be re-established and UPR-associated cell death programs may be also executed [3–5].

It is widely accepted that the UPR is hyperactivated in cancer that reflects cancer cell adaptations to intrinsic (e.g., high demand for protein synthesis) and extrinsic (e.g., restricted supply of nutrients and oxygen, and chemotherapy) ER stress conditions [6, 7]. The UPR may contribute to several hallmarks of cancer such as genome instability, sustained proliferation, cell death and chemotherapy resistance, angiogenesis induction, immune suppression, inflammation, invasion and metastasis [7, 8]. Perhaps this may be partly achieved by the interactions of the UPR sensors with newly identified binding partners as a part of a multi-protein complex named the UPRosome with novel UPR functions in diverse biological processes such as cytoskeleton dynamics, mitochondrial bioenergetics, cell differentiation, autophagy and DNA damage and repair [9–11]. Thus, targeting the ER stress signaling components seems to be a promising anticancer strategy [6, 8, 10, 11].

DNA methyltransferase 2/tRNA aspartic acid methyltransferase 1 (DNMT2/TRDMT1) may modulate a number of stress responses, cell proliferation and survival in normal and cancer cells [12–20]. The role of DNMT2/TRDMT1-mediated RNA C-5 methylation in maintaining tRNA and genetic stability and in promoting protein synthesis was proposed [18, 21–25]. For example, in *Dnmt2* wild-type mice, C38 tRNA methylation secured the fidelity of tRNA(Glu) and tRNA(Asp) codons that promoted the translation of correctly folded proteins [24]. In contrast, in *Dnmt2*^−^/^−^ mice, the lack of C38 tRNA methylation compromised the capacity of tRNAs to properly discriminate between Asp and Glu codons that resulted in diminished translational fidelity [24]. The authors suggested that the loss of Dnmt2 may promote the production of unfolded proteins and an aberrant phenotype in mice [24]. However, the effect of *DNMT2/TRDMT1* gene knockout on ER proteostasis and the UPR was not directly addressed. This seems to be particularly interesting in the case of cancer cells with highly active UPR with pro-survival and oncogenic functions [6–8].

In the present study, four genetically distinct cellular cancer models (breast and cervical cancer, osteosarcoma and glioblastoma cells) were utilized to investigate the impact of *DNMT2/TRDMT1* gene knockout on doxorubicin (DOX)-induced ER stress and the UPR. Tunicamycin (TUN), a well-established inducer of ER stress, was also used for comparison. We have shown for the first time that the loss of *DNMT2/TRDMT1* gene attenuated DOX-associated PERK activation and impaired NSUN-mediated responses that was accompanied by decreased pools of miR-23a-3p, miR-93-5p, miR-125a-5p and miR-191-5p controlling protein turnover and translational regulation. When ER stress was prolonged, cells with *DNMT2/TRDMT1* gene knockout were more sensitive to apoptotic cell death than corresponding control cells.

## Materials and methods

### Cancer cell lines with *DNMT2/TRDMT1* gene knockout

Four human cancer cell lines (MDA-MB-231 breast cancer cells, HeLa cervical cancer cells, U-2 OS osteosarcoma cells, U-251 MG glioblastoma cells) with CRISPR-based *DNMT2/TRDMT1* gene knockout were obtained and cultured as described elsewhere [19]. The stability of *DNMT2/TRDMT1* gene knockout was routinely checked using anti-DNMT2 antibody (A-7, sc-271513, Santa Cruz Biotechnology, Dallas, TX, USA) and western blotting procedure [16]. Two types of plasmid containing cells were considered, namely control cells containing control plasmid (C-NIC cells, control double nickase cells) and cells with *DNMT2/TRDMT1* gene knockout (D-NIC cells, DNMT2/TRDMT1 double nickase cells) [19].

### Doxorubicin and tunicamycin treatments

Doxorubicin hydrochloride (DOX, 44583) and tunicamycin (TUN, 654380) were purchased from Merck KGaA (Darmstadt, Germany). To select DOX concentration inducing a moderate cytotoxic effect, C-NIC and D-NIC cells were treated with 0.1, 0.25, 0.5, 1 and 2 µM DOX for 24 h and MTT test was then assayed [15]. The concentration of 1 µM DOX that lowered the metabolic activity (the activity of mitochondrial dehydrogenases) of four cancer cell lines of about 10–30% compared to control conditions was selected for further analysis. Apoptotic activity of DOX was also evaluated for prolonged treatment for 48 h with 35 nM (senescence inducing concentration) [19] and 1 µM DOX. The treatment with 10 µg/ml TUN for 24 h and/or 48 h (an inhibitor of protein *N*-glycosylation and an inductor of ER stress) was used as a positive control for the induction of UPR [26].

### Cell proliferation and DNA fragmentation: imaging flow cytometry

As a marker of cell proliferation, Ki67 immunostaining was considered. Muse™ Ki67 Proliferation Kit was used (MCH100114, Luminex Corporation, Austin, TX, USA). Apoptosis-associated DNA strand breaks were revealed using TUNEL (TdT-mediated dUTP-X nick end labeling) assay (11684795910, *In Situ* Cell Death Detection Kit, Fluorescein, Roche, Basel, Switzerland). Digital cell images were captured and Ki67-positive cell population and DNA damage-positive cell population were analyzed using an Amnis® FlowSight® Imaging Flow Cytometer and IDEAS software version 6.2.187.0 (Luminex Corporation, Austin, TX, USA). Representative histograms are presented.

### Cytotoxicity

DOX-mediated apoptosis was investigated after 48 h treatment with DOX at two concentrations of 35 nM and 1 µM, whereas pro-apoptotic activity of 10 µg/ml TUN was evaluated after 24 and 48 h stimulation. Two apoptotic biomarkers were considered, namely phosphatidylserine externalization and pan-caspase activity. Muse® Cell Analyzer, Muse® Annexin V and Dead Cell Assay Kit and Muse® Multi-caspase Assay Kit were used according to manufacturer’s instructions (Luminex Corporation, Austin, TX, USA).

### Protein carbonylation

Protein carbonylation was evaluated using an OxyBlot™ Protein Oxidation Detection Kit (S7150, Merck KGaA, Darmstadt, Germany) and the protocol according to the manufacturer’s instructions [16]. OxyBlot™ Protein Standard [a mixture of proteins containing 2,4-dinitrophenylhydrazone (DNP) residues] was also used.

### Protein aggregation

Protein aggregation was evaluated using PROTEOSTAT® Protein Aggregation Kit (ENZ-51023, Enzo Life Sciences, Inc., Farmingdale, NY, USA) and the protocol according to the manufacturer’s instructions. Protein aggregation is presented as relative fluorescence units (RFU).

### Proteasomal activity

Proteasomal activity was assayed using Fluorogenic Proteasome Substrate III (Suc-Leu-Leu-Val-Tyr-AMC, chymotrypsin-like activity, 539142, Merck KGaA, Darmstadt, Germany). Briefly, 2 × 10^6^ of cells were used to determine proteasomal activity. The final substrate concentration was 100 µM and the fluorescence (λ_ex_ = 380 nm; λ_em_ = 460 nm) was measured in a Tecan Infinite® M200 microplate fluorescence reader. The protocol was applied according to Chen et al. [27]. Proteasomal activity is presented as relative fluorescence units (RFU).

### Immunofluorescence and F-actin staining

Cell fixation and immunostaining were conducted as previously reported [28]. eIF4E was detected using anti-eIF4E primary antibody (1:100, MA1-089) and secondary antibody conjugated to Cy5 (1:1000, A10524) (Thermo Fisher Scientific, Waltham, MA, USA). Filamentous actin (F-actin) was revealed using Alexa Fluor™ 488 Phalloidin staining (A12379, Thermo Fisher Scientific, Waltham, MA, USA). Nucleic acids were visualized using acridine orange staining and Cy3 filter. Monochromatic digital cell images were captured using a laser-based confocal imaging and HCA system IN Cell Analyzer 6500 HS (Cytiva, Marlborough, MA, USA) and presented using RGB color system. eIF4E signals are presented in red, F-actin signals in green and nucleic acids in blue. Quantitative analysis was conducted using IN Carta software (Cytiva, Marlborough, MA, USA). The immunofluorescent signals of eIF4E (eIF4E levels) are presented as RFUs. Stress granules (puncta with eIF4E immunosignals) were calculated per cell. F-actin cytoskeleton was analyzed using two parameters, namely average fiber length and F-actin heterogeneity.

### Western blotting

Upon DOX and/or TUN treatment, proteins were extracted and western blotting protocol was conducted as previously described [29]. The following primary and secondary antibodies were used, namely, anti-caspase 3 (1:4000, PA5-77887), anti-HSP90 (1:1000, MA1-10373), anti-SOD1 (1:3000, PA5-23245), anti-LC3B (1:1000, ab51520), anti-AKT (1:1000, 4691), anti-phospho-AKT (Ser473) (1:500, 4060), anti-AMPK (1:1000, PA5-14026), anti-phospho-AMPK (Thr183, Thr172) (1:1000, PA5-17831), anti-GRP78 (1:5000, PA5-85169), anti-ATF6 (1:500, PA5-68556), anti-PERK (1:500, PA5-79193), anti-phospho-PERK (Thr980) (1:750, MA5-15033), anti-eIF2α (1:1000, AHO1182), anti-phospho-eIF2α (Ser52) (1:1000, 44-728G), anti-IRE1 (1:1000, PA5-20190), anti-phospho-IRE1 (Ser724) (1:1000, PA1-16927), NSUN1 (1:3000, PA5-59073), NSUN2 (1:1000, 702036), NSUN3 (1:2000, PA5-57561), NSUN4 (1:1000, PA5-31695), NSUN5 (1:1500, PA5-54228), NSUN6 (1:3000, PA5-61119), anti-β-actin-peroxidase (1:40000, A3854), anti-mouse IgG HRP-linked antibody (1:3000, 7076) and anti-rabbit IgG HRP-linked antibody (1:3000, 7074) (Thermo Fisher Scientific, Waltham, MA, USA, Merck KGaA, Darmstadt, Germany, Abcam, Cambridge, UK and Cell Signaling Technology, Danvers, MA, USA). The results represent the relative density normalized to β-actin (loading control). Phospho-AKT, phospho-AMPK, phospho-PERK, phospho-eIF2α and phospho-IRE1 immunosignals were normalized to AKT, AMPK, PERK, eIF2α and IRE1 immunosignals, respectively. The activation of caspase 3 was analyzed as a ratio of cleaved caspase 3 to pro-caspase 3 (full length). The activation of ATF6 was investigated as a ratio of cleaved form (p50ATF6) to β-actin.

### MicroRNA expression patterns and target analysis

RNA was isolated using GenElute™ Mammalian Total RNA Miniprep Kit (RTN70, Merck KGaA, Darmstadt, Germany) according to the manufacturer’s instructions. The purity and quantity of RNA was assayed using NanoDrop™ 2000 UV–Vis microvolume spectrophotometer (Thermo Fisher Scientific, Waltham, MA, USA). MicroRNA profiling was conducted using nCounter® Analysis System and nCounter Human v2 miRNA Panel containing 798 unique microRNA barcodes (NanoString Technologies, Seattle, WA, USA) according to previously reported protocol [30]. MicroRNA data analysis was performed using nSolver 4.0 analysis software (NanoString Technologies, Seattle, WA, USA). Background correction was performed by calculating background threshold of a negative control (≤ 100 count value) as a cut-off. Positive control was normalized using a geometric mean. For technical variations, code-set content was performed with a geometric mean for housekeeping genes. Stability analysis of expressed miRNAs was performed for miRNA with average counts above background. For estimation of fold changes, all pairwise ratios were used. The hierarchical clustering of microRNA expression data was performed using Genesis 1.8.1 [31]. The average group linkage UPGMA was used to generate a heatmap. Venn diagram (http://bioinformatics.psb.ugent.be/webtools/Venn/) was used to calculate the intersection between up- or down-regulated microRNAs in HeLa, MDA-MB-231, U-2 OS and U-251 MG cells with *DNMT2/TRDMT1* gene knockout (D-NIC cells) compared to control cells (C-NIC cells) as well as between D-NIC in control conditions and DOX-treated D-NIC cells. Identified microRNAs with affected levels in four cancer cell lines were subjected to gene and pathway target analysis using DIANA-miRPath v3.0 online software [32]. To visualize microRNA gene networks (a total number of genes = 1947 for C-NIC cells versus D-NIC cells and a total number of genes = 1713 for D-NIC cells in control conditions versus DOX-treated D-NIC cells), the visual analytics platform miRNet and database miRTarBase v8.0 were used. Enriched horizontal bars with colors were created using WeiShengxin (http://www.bioinformatics.com.cn) on the basis of data generated with DIANA tools. Observed changes in the levels of selected microRNA in four cancer cells were then validated using the RT-PCR assay. Briefly, RT-PCR was applied according to miRCURY® LNA® miRNA PCR manual (QIAGEN, Germantown, MD, USA). The miRCURY LNA RT kit (QIAGEN, Germantown, MD, USA) was used for reverse transcription reaction according to the manufacturer’s instructions. The miRCURY SYBR Green PCR Kit with ROX dye and miRCURY LNA primers for detection of selected microRNAs, such as: hsa-let-7a-5p, hsa-let-7b-5p, hsa-let-7i-5p, hsa-miR-181a-5p, hsa-miR-23a-3p, hsa-miR-93-5p, hsa-miR-125a-5p and hsa-miR-191-5p, were used according to the manufacturer’s instructions (QIAGEN, Germantown, MD, USA). The U6 snRNA was considered as a reference gene. The results were quantified and compared to corresponding cells with active *DNMT2/TRDMT1* gene (C-NIC) under control conditions.

### Statistical analysis

The results represent the mean ± SD from at least three independent experiments. Box and whisker plots with median, lowest, and highest values were also considered. Differences between control C-NIC cells and other samples were analyzed using one-way ANOVA and Dunnett’s multiple comparison test, whereas differences between C-NIC cells and D-NIC cells were revealed using one-way ANOVA and Tukey’s multiple comparison test. Statistical significance was evaluated using GraphPad Prism 5. *P*-values of less than 0.05 were considered significant.

## Results and discussion

### The effect of *DNMT2/TRDMT1* gene knockout on DOX-mediated cytotoxicity and oxidative stress

**Fig. 1:**
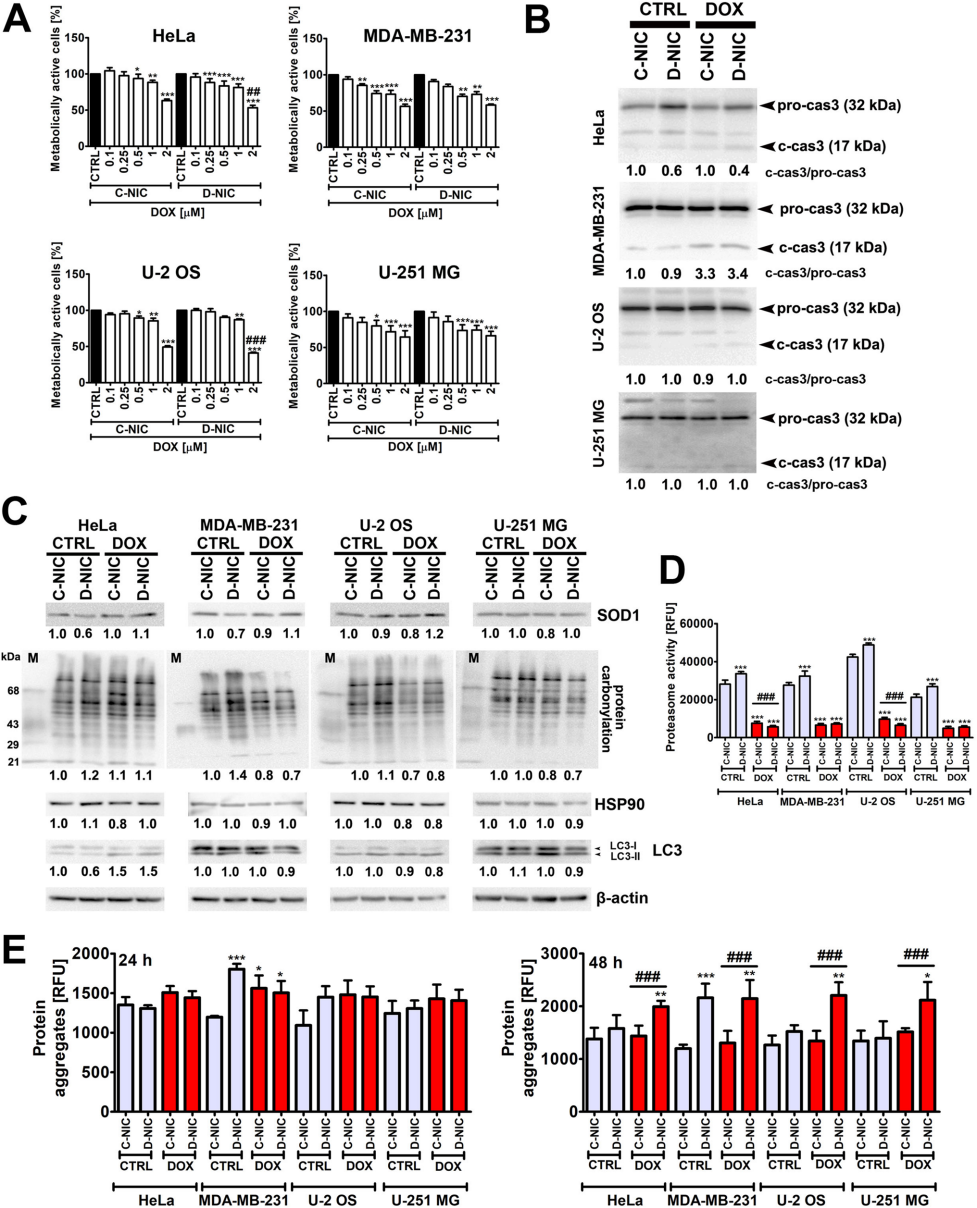
Doxorubicin (DOX)-mediated changes in metabolic activity (**A**), caspase 3 activation (**B**), oxidative stress and autophagy (**C**), proteasomal activity (**D**) and protein aggregation (**E**) in four cancer cell lines (HeLa cervical cancer cells, MDA-MB-231 breast cancer cells, U-2 OS osteosarcoma cells and U-251 MG glioblastoma cells) lacking active *DNMT2/TRDMT1* gene (D-NIC cells). **A** Metabolic activity was assessed using MTT assay. Cells were incubated with 0.1, 0.25, 0.5, 1 and 2 µM DOX for 24 h and the concentration of 1 µM was selected for further analyses. Metabolic activity at control conditions (CTRL) is considered as 100%. **B** Western blot-based analysis of caspase 3 cleavage (activation). Full length (non-active) caspase 3 and cleaved (active) caspase 3 were analyzed. Caspase 3 activity is presented as a ratio of cleaved caspase to full length caspase 3. **C** Western blot-based analysis of the levels of SOD1, protein carbonylation, HSP90 and a lipidated form of LC3 (LC3-II). For the evaluation of oxidative protein damage (protein carbonylation), OXYBLOT assay was used. Lane M denotes a positive control, namely a mixture of proteins containing 2,4-dinitrophenylhydrazone (DNP) residues. β-Actin antibody was considered as a loading control. Data were normalized to C-NIC untreated control. **D** Chymotrypsin-like proteasomal activity was assayed using fluorogenic proteasome substrate Suc-Leu-Leu-Val-Tyr-AMC. Proteasomal activity is presented as relative fluorescence units (RFU). **E** Protein aggregation was evaluated using PROTEOSTAT® Protein Aggregation Kit. Protein aggregation is presented as relative fluorescence units (RFU). Two time points were considered, namely 24 h (left) and 48 h (right) treatment with 1 µM DOX. Bars indicate SD, n = 3, ***p < 0.001, **p < 0.01, *p < 0.05 compared to untreated C-NIC cells (ANOVA and Dunnett’s a posteriori test), ^###^*p* < 0.001, ^##^*p* < 0.01 compared to drug-treated C-NIC cells (ANOVA and Tukey’s a posteriori test). *CTRL* control conditions, *DOX* doxorubicin treatment, *C-NIC* control cells with unaffected levels of DNMT2/TRDMT1, *D-NIC* cells with *DNMT2/TRDMT1* gene knockout

ER stress can be considered as a one of mechanisms for DOX-mediated side-effects during chemotherapy, namely cardiotoxicity [33]. However, DOX-treated MCF-7 breast cancer cells may be also subjected to ER stress-mediated cell death [34]. Treatment with 10 µM DOX for 24 h resulted in a decrease in cell viability to 50% of control levels and elevated reactive oxygen species (ROS) production in MCF-7 breast cancer cells [34]. ER stress signaling was activated as early as 3 h upon DOX stimulation (increased levels of DDIT3/CHOP) and at 12 and 24 h of treatment (increased levels of ATF6) that was accompanied by elevated levels of pro-apoptotic Bax and diminished levels of anti-apoptotic Bcl-xL at 12 h of exposure [34]. In our experimental conditions, DOX, when used at the concentration of 2 µM for 24 h, decreased metabolic activity of cancer cells with unmodified levels of DNMT2/TRDMT1 (C-NIC cells) to 49% (U-2 OS osteosarcoma cells), 56% (MDA-MB-231 breast cancer cells), 63% (HeLa cervical cancer cells) and 64% (U-251 MG glioblastoma cells) of control levels at control conditions (Fig. 1A).

In the case of U-2 OS (a decrease in metabolic activity to 41% of control levels) and HeLa cells (a decrease in metabolic activity to 53% of control levels), *DNMT2/TRDMT1* gene knockout sensitized D-NIC cells to 2 µM DOX stimulation compared to corresponding C-NIC cells (Fig. 1A). As we did not intend to induce massive cytotoxicity that perhaps would mask DOX-stimulated ER stress and the effect of *DNMT2/TRDMT1* gene knockout, we decided to use the concentration of 1 µM DOX and 24 h stimulation for further analyses. Treatment with 1 µM DOX resulted in a decrease in metabolic activity of about 12–28% compared to control conditions in four cancer cell lines (Fig. 1A). The effect of DOX on Ki67 expression was mild in four cancer cell lines (Fig. S1A). Also *DNMT2/TRDMT1* gene knockout did not result in significant changes in Ki67 expression (Fig. S1A). Thus, DOX, when used at the concentration of 1 µM for 24 h, did not affect cell proliferation as judged by Ki67 immunostaining (Fig. S1A). However, we have previously reported that DOX at senescence inducing concentration (35 nM that is almost 30 times lower concentration than the concentration used in the present study), when treated for 24 h, promoted G2/M cell cycle arrest of the cell cycle of HeLa, MDA-MB-231, U-2 OS and U-251 MG cells [19]. Moreover, *DNMT2/TRDMT1* gene knockout potentiated this effect in MDA-MB-231 cells and U-251 MG cells [19]. Perhaps Ki67 expression (this study) is not correlated with DOX-mediated perturbations in cell cycle progression for short time treatment [19]. DOX-associated changes in metabolic activity (Fig. 1A) was also accompanied by apoptosis induction in MDA-MB-231 cells (Fig. 1B; Fig. S1B). 1 µM DOX treatment promoted DNA fragmentation and caspase 3 cleavage that was more apparent in MDA-MB-231 D-NIC cells than MDA-MB-231 C-NIC cells (Fig. 1B; Fig. S1B). MDA-MB-231 D-NIC cells were also more prone to 35 nM DOX-induced apoptosis compared to MDA-MB-231 C-NIC cells as judged by Annexin V staining [19]. However, when the treatments with DOX at senescence inducing concentration (35 nM) [19] and at 1 µM (this study) were prolonged to 48 h (Fig. S2), more severe cytotoxicity was observed. Except of U-2 OS D-NIC cells, cancer cells lacking active *DNMT2/TRDMT1* gene were more prone to 35 nM DOX treatment (Fig. S2A). After 48 treatment with 1 µM DOX, a majority of C-NIC and D-NIC cells were dead (Fig. S2A, B). It has been previously reported that breast cancer cell response to 1 µM DOX treatment may be complex [35]. As a primary response, apoptotic cell death is initiated [35]. However, senescence program (p21 upregulation, downregulation of cdc2 and increased ROS production) is executed in the residual surviving population regardless of p53 status [35]. *DNMT2/TRDMT1* gene knockout also potentiated apoptotic cell death in DOX-induced senescent HeLa and MDA-MB-231 D-NIC cells compared to corresponding DOX-induced senescent C-NIC cells [19]. On the other hand, suppression of apoptosis in DOX-treated MDA-MB-231 and MCF-7/E6 cells, resulted in substantial induction of cytotoxic autophagy [35]. The authors concluded that accelerated senescence, autophagy and apoptosis all appear to be effective in suppressing self-renewal capacity in breast cancer cells stimulated with DOX [35].

It is widely accepted that increased ROS production is implicated in DOX anti-cancer action [35–38]. For example, DOX-stimulated oxidative stress promoted apoptotic cell death in diverse cancer cells [37, 38]. More recently, we have also shown that ROS production is increased in DOX-induced senescent HeLa, MDA-MB-231, U-2 OS and U-251 MG cells and *DNMT2/TRDMT1* gene knockout intensified oxidative stress in HeLa and U-251 MG D-NIC cells [19]. Low molecular weight antioxidants glutathione (GSH) and *N*-acetyl cysteine (NAC) may diminish DOX-induced senescence program in MCF-7 and MDA-MB-231 breast cancer cells [35]. However, DOX-mediated ROS levels may also promote opposite effects that perhaps reflect considered concentrations [39]. For example, a subclinical concentration of 100 nM DOX stimulated ROS-mediated invasive activity of U-2 OS osteosarcoma cells [39]. As ER stress and the UPR may be accompanied or mediated by redox imbalance [40–42], we have decided then to analyze the levels of superoxide dismutase 1 (SOD1), a part of oxidative stress response and oxidative protein damage, here protein carbonylation, upon stimulation with 1 µM DOX (Fig. 1C). DOX treatment did not result in a significant increase in SOD1 levels and protein carbonylation in four cancer cell lines (Fig. 1C). However, MDA-MB-231 and HeLa cells with *DNMT2/TRDMT1* gene knockout were slightly more prone to oxidative protein damage than corresponding C-NIC cells in control conditions (Fig. 1C). DOX treatment also did not promote HSP90-based adaptive response in four cancer cells (Fig. 1C). It was documented that ROS resistance in breast cancer cells is accompanied by a constitutive overexpression of the endoplasmic reticulum chaperone, GRP94, whereas levels of its cytoplasmic homolog HSP90, or GRP78, were not affected [43]. This effect was not mediated by constitutive UPR activation [43]. As GRP94 overexpression promoted cell proliferation and migration in ROS resistant cancer cells, elevated levels of GRP94 were proposed as a hallmark of aggressiveness in breast cancers [43].

### The effect of *DNMT2/TRDMT1* gene knockout on DOX-mediated changes in protein degradation pathways

We have next asked the question of whether *DNMT2/TRDMT1* gene knockout may affect two major pathways for intracellular protein degradation (Fig. 1C, D). Autophagic pathway in cancer cells lacking active *DNMT2/TRDMT1* gene was not modified compared to corresponding C-NIC cells as judged by unaffected levels of LC3 in control conditions (Fig. 1C). Except of DOX-mediated increase in the levels of LC3-II in HeLa cells both C-NIC and D-NIC cells, 1 µM DOX treatment did not induce autophagy in MDA-MB-231, U-2 OS and U-251 MG cells (Fig. 1C). However, DOX-mediated autophagic response in cancer cells may be concentration-dependent. Indeed, DOX at senescence inducing concentration (35 nM) promoted an elevation in the levels of LC3 in HeLa, MDA-MB-231, U-2 OS and U-251 MG cells [19]. Moreover, DOX-induced autophagy in different cancer cells may exert both cytoprotective and cytotoxic effects [35, 44]. Pharmacological inhibition of apoptosis (ZVAD-fmk) resulted in cytotoxic autophagy in DOX-treated breast cancer cells, whereas DOX-induced apoptosis was blocked by the induction of cytoprotective autophagy in osteosarcoma cells [35, 44]. Disruption of cytoprotective autophagy by the means of genetic (ATG7 siRNA) or pharmacological (3‑methyladenine) inhibitors sensitized osteosarcoma cells to DOX-induced apoptosis [43].

In contrast, an increase of about 20% in proteasomal activity (chymotrypsin-like activity) was observed in four cancer cell lines with *DNMT2/TRDMT1* gene knockout in control conditions (*p* < 0.001, Fig. 1D). DOX treatment resulted in diminished proteasomal activity in four cancer cell lines (*p* < 0.001, Fig. 1D). Moreover, in DOX-treated HeLa and U-2 OS D-NIC cells, a decrease in proteasomal activity was more pronounced compared to corresponding DOX-treated C-NIC cells (Fig. 1D). Proteasomal activity of DOX-treated HeLa and U-2 OS D-NIC cells was lowered of 25% and 34% compared to corresponding DOX-treated C-NIC cells, respectively (*p* < 0.001, Fig. 1D). This may suggest that *DNMT2/TRDMT1* gene knockout may compromise the action of the ubiquitin-proteasome system (UPS) in DOX-treated HeLa and U-2 OS D-NIC cells (Fig. 1D). Selected proteasome inhibitors such as MG132, ixazomib, carfilzomib, delanzomib and bortezomib were reported to sensitize cervical and breast cancer cells, and osteosarcoma and glioblastoma cells to DOX-induced apoptosis [45–49]. For example, bortezomib potentiated DOX-mediated anti-cancer action through ROS-dependent activation of peIF2α/ATF4/CHOP axis in osteosarcoma cells [49]. DOX-promoted decrease in proteasomal activity did not correlate with massive protein aggregation in four cancer cells after 24 h treatment (Fig. 1E). Except of DOX-treated MDA-MB-231 cells (*p* < 0.05), DOX-induced mild increase in the levels of protein aggregates in HeLa, U-2 OS and U-251 MG cells that was of no statistical significance (Fig. 1E). In contrast, *DNMT2/TRDMT1* gene knockout in MDA-MB-231 D-NIC cells resulted in 50% increase in protein aggregation compared to corresponding C-NIC cells in control conditions (*p* < 0.001, Fig. 1E). *DNMT2/TRDMT1* gene knockout did not potentiate the formation of protein aggregates after treatment with DOX for 24 h (Fig. 1E). However, when the exposure to DOX was prolonged (48 h stimulation), one can observe DOX-mediated increase in the levels of protein aggregates in four cancer cell lines lacking active *DNMT2/TRDMT1* gene (Fig. 1E). Indeed, this suggests that *DNMT2/TRDMT1* gene knockout affects protein homeostasis upon prolonged treatment with DOX that may result in increased sensitivity to DOX upon 48 h stimulation (Fig. S2).

### *DNMT2/TRDMT1* gene knockout suppresses DOX-mediated activation of PERK-based branch of the UPR in three cancer cell lines

**Fig. 2:**
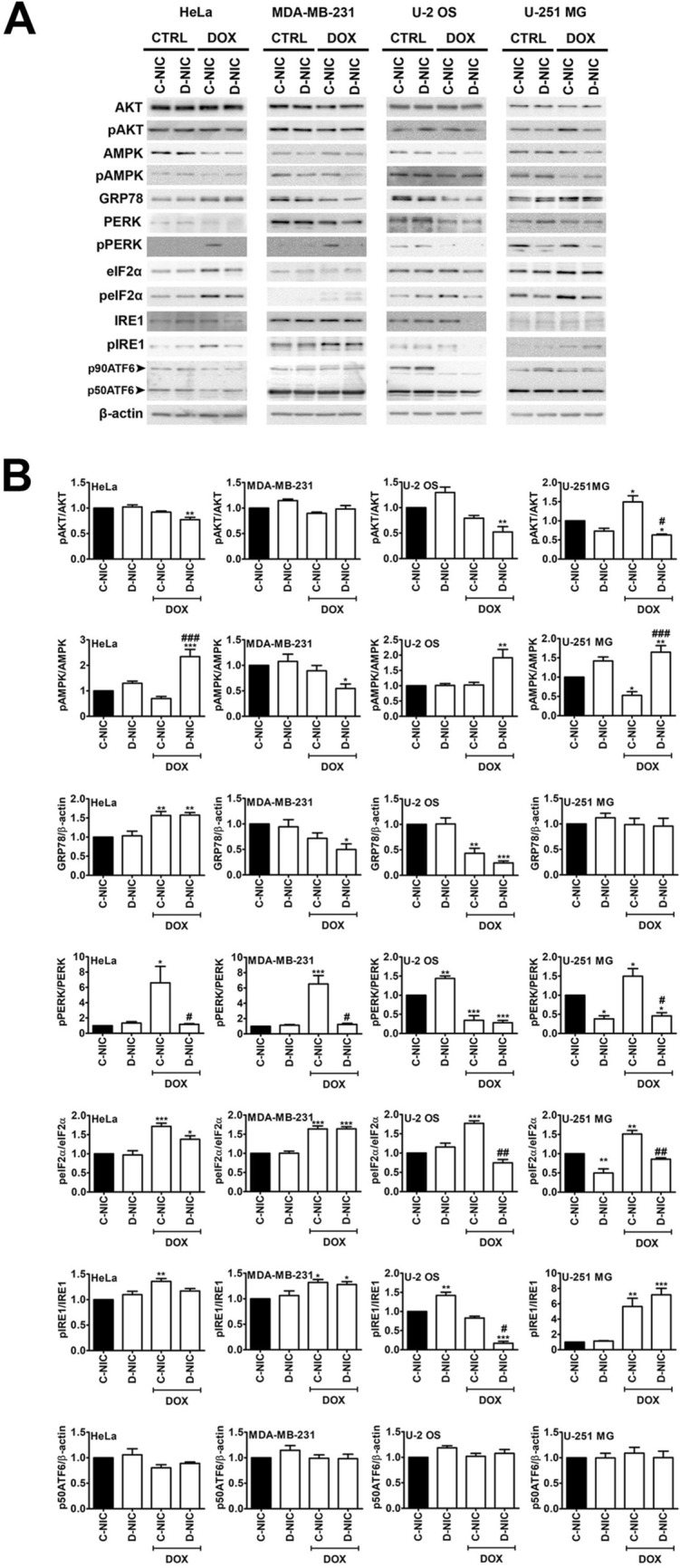
The effect of *DNMT2/TRDMT1* gene knockout on DOX-mediated changes in the activity of AKT and AMPK pathways and the UPR in four cancer cell lines. **A** Western blot-based analysis of the levels of AKT, phosphorylated AKT, AMPK, phosphorylated AMPK, GRP78, PERK, phosphorylated PERK, eIF2α, phosphorylated eIF2α, IRE1, phosphorylated IRE1 and p50ATF6. β-Actin antibody was considered as a loading control. **B** Data were normalized to C-NIC untreated control. The activity of AKT, AMPK, PERK, eIF2α and IRE1 was analyzed as a ratio of phosphorylated AKT to AKT, phosphorylated AMPK to AMPK, phosphorylated PERK to PERK, phosphorylated eIF2α to eIF2α and phosphorylated IRE1 to IRE1, respectively. The activity of ATF6 was analyzed as an occurrence of cleaved form of ATF6, namely p50ATF6. Bars indicate SD, n = 3, ***p < 0.001, **p < 0.01, *p < 0.05 compared to untreated C-NIC cells (ANOVA and Dunnett’s *a posteriori* test), ^###^*p* < 0.001, ^#^*p* < 0.05 compared to drug-treated C-NIC cells (ANOVA and Tukey’s a posteriori test). *CTRL* control conditions, *DOX* doxorubicin treatment, *C-NIC* control cells with unaffected levels of DNMT2/TRDMT1, *D-NIC* cells with *DNMT2/TRDMT1* gene knockout

More recently, it was shown that 5 µM DOX and 24 h treatment can activate ER stress-dependent cell death effectors leading to apoptotic cell death in drug-sensitive osteosarcoma cells [50]. Moreover, 5 µM H_2_S‑releasing doxorubicin (SDOX) can also sensitize drug-resistant osteosarcoma cells to ER-dependent apoptosis that is achieved by preferential accumulation of SDOX in the ER lumen, SDOX-mediated sulfhydration and concomitant ubiquitination of ER-associated proteins and decline in the levels of the drug efflux transporter ABCB1/P-glycoprotein (Pgp) [50]. The authors concluded that both DOX and SDOX stimulated the same ER-dependent pro-apoptotic pathways, but different upstream mechanisms were involved, namely DOX-mediated oxidative stress and SDOX-mediated reductive stress in drug-sensitive and drug-resistant osteosarcoma cells, respectively [50]. 10 µM DOX and 24 h treatment also promoted ER stress in MCF-7 breast cancer cells that resulted in the activation of DDIT3/CHOP-dependent Bax pathway leading to apoptotic cell death [34]. In the present study, we have decided to induce mild ER stress by using 1 µM DOX for 24 h and investigate the effects in four cancer cell lines lacking functional *DNMT2/TRDMT1* gene (Fig. 2).

As the 78-kDa glucose-regulated protein (GRP78, BiP or HSPA5), a master regulator for ER stress, is a major ER chaperone with the antiapoptotic properties and the ability to control the activation of UPR [51], first, we have analyzed its levels upon DOX stimulation and *DNMT2/TRDMT1* gene knockout (Fig. 2). When unfolded/misfolded proteins are accumulated in the ER lumen, GRP78 is sequestered away from three major ER sensors (PERK, IRE1 and ATF6) that enables PERK and IRE1 activation through homodimerization and autophosphorylation and ATF6 activation through translocation from the ER to the Golgi apparatus followed by protease-mediated fragmentation [51]. GRP78 levels were elevated in DOX-treated HeLa cells and decreased in DOX-treated MDA-MB-231 and U-2 OS cells compared to control conditions (Fig. 2). No effect of *DNMT2/TRDMT1* gene knockout was observed (Fig. 2). GRP78 overexpression is commonly observed in cancer cell lines that is associated with aggressive growth and invasive properties [52]. It was reported that GRP78/BiP and PERK coordinated p38 signaling-mediated survival response against DOX-induced apoptosis by preventing Bax activation in dormant HEp3 squamous carcinoma cells [53]. As GRP78 can be both an upstream regulator of the PI3K-AKT oncogenic signaling pathway and a downstream target of AKT activation [52], we have also analyzed the phosphorylation status of AKT and found that decreased levels of GRP78 were correlated with diminished phospho-AKT signals in DOX-treated MDA-MB-231 and U-2 OS D-NIC cells (Fig. 2). Targeting GRP78 by anti-GRP78 monoclonal antibody resulted in the suppression of PI3K/AKT signaling, tumor growth and metastasis [54]. Several ER stress inducers promoted a cytosolic ataxia telangiectasia mutated (ATM)-AKT-GSK3β-αNAC/γTX signaling axis [55]. Cytosolic ATM acted like a platform, where protein phosphatase 2 A (PP2A) dephosphorylated AKT leading to mitochondria-dependent apoptosis [55]. In contrast, DOX promoted AMPK activation in HeLa and U-2 OS D-NIC cells (Fig. 2) that can be considered as a sign of energy metabolic stress [56]. However, AMPK activation in DOX-treated HeLa and U-2 OS D-NIC cells did not promote the activity of autophagic pathway (Fig. 1C). AMPK is also reported to be a physiological suppressor of the UPR in cancer cells [57]. AMPK knockdown induced ER stress and sensitized acute lymphoblastic leukemia (ALL) cells to combined treatment of AICA riboside and methotrexate [57]. In contrast, AKT inhibition, a cellular antagonist of AMPK, promoted AMPK activity and attenuated ER stress-mediated apoptosis in ALL cells [57].

Three UPR arms were then considered, namely PERK-, IRE1- and ATF6-mediated signaling (Fig. 2). 1 µM DOX mainly induced PERK-based part of the UPR as judged by increased levels of phosphorylated form of PERK in HeLa, MDA-MB-231 and U-251 MG C-NIC cells (Fig. 2). PERK activation was not observed in D-NIC cells with dysfunctional *DNMT2/TRDMT1* gene that in the case of DOX-treated HeLa D-NIC cells may be due to increased activity of AMPK, a suppressor of the UPR (Fig. 2). In the case of U-2 OS cells, no DOX-mediated PERK activation was noticed in both C-NIC and D-NIC cells (Fig. 2). PERK silencing limited glioma cell viability and ATP/lactate production during low glucose stress that was mediated by attenuated AKT activation and subsequent inhibition of mitochondrial translocation of hexokinase II (HK2) [58]. PERK/Nrf2/MRP1 axis also conferred resistance to ER stress- and chemotherapy-induced cell death in colon cancer cells [59].

**Fig. 3:**
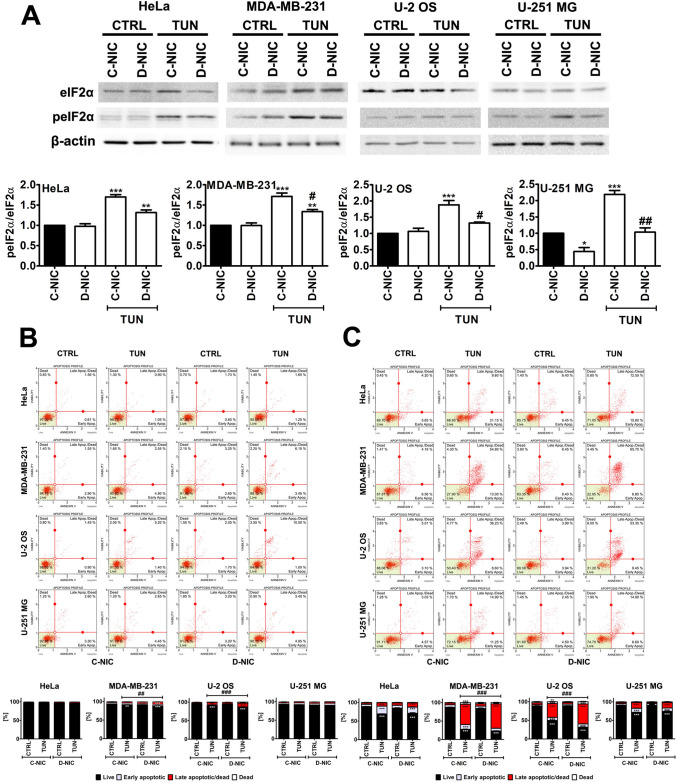
The effect of *DNMT2/TRDMT1* gene knockout on TUN-mediated changes in the activity of eIF2α (**A**) and apoptosis induction (**B**, **C**) in four cancer cell lines. **A** After 24 h treatment with 10 µg/ml TUN, the activity eIF2α was analyzed as a ratio of phosphorylated eIF2α to eIF2α using western blotting. After 24 h (**B**) and 48 h (**C**) treatment with 10 µg/ml TUN, apoptosis induction was evaluated using flow cytometry and Annexin V staining. Representative dot plots are presented. Bars indicate SD, n = 3, ***p < 0.001, **p < 0.01, *p < 0.05 compared to untreated C-NIC cells (ANOVA and Dunnett’s a posteriori test), ^###^*p* < 0.001, ^##^*p* < 0.01, ^#^*p* < 0.05 compared to drug-treated C-NIC cells (ANOVA and Tukey’s a posteriori test). *CTRL* control conditions, *TUN* tunicamycin treatment, *C-NIC* control cells with unaffected levels of DNMT2/TRDMT1, *D-NIC* cells with *DNMT2/TRDMT1* gene knockout

PERK activation was accompanied by increased phosphorylation status of eIF2α in HeLa and U-251 MG C-NIC cells (Fig. 2). However, eIF2α activation was also observed in DOX-treated U-2 OS C-NIC cells with no PERK activation (Fig. 2). Thus, in some cellular settings, eIF2α activation can be considered as PERK-independent. *DNMT2/TRDMT1* gene knockout in U-2 OS and U-251 MG cells compromised eIF2α activation as judged by decreased signals of phosphorylated eIF2α in D-NIC cells compared to C-NIC cells after DOX stimulation (Fig. 2). We have previously shown that 5-azacytidine, a methyltransferase inhibitor, provoked eIF2α-based response in oxidant-stressed mouse insulinoma β-TC-6 cells that was mediated by elevated levels of Trdmt1 [60]. However, 5-azacytidine treatment resulted in functional inactivation of Trdmt1 and cell elimination by the means of cytotoxic autophagy [60]. In the present study, *DNMT2/TRDMT1* gene knockout also affected DOX-induced UPR signaling and ER stress leading to apoptotic cell death when cancer cells were incubated with ER stress inducer for 48 h (Fig. S2). DOX-mediated stimulation of IRE1 activity was the most pronounced in U-251 MG C-NIC and D-NIC cells and to a lesser extent, in HeLa and MDA-MB-231 C-NIC and D-NIC cells (Fig. 2). In contrast, *DNMT2/TRDMT1* gene knockout in U-2 OS cells resulted in non-detectable levels of IRE1 and phosphorylated IRE1 in DOX-treated D-NIC cells (Fig. 2). Surprisingly, ATF6 activation (protease-mediated fragmentation) was observed in four cancer cell lines in control conditions (Fig. 2). *DNMT2/TRDMT1* gene knockout did not affect ATF6 activation (Fig. 2). DOX treatment did not potentiate ATF6 activation in four cancer cell lines (Fig. 2). Elevated expression of ATF6 and cancerous inhibitor of protein phosphatase 2 A (CIP2A, an oncogene) is correlated with poor prognosis of colon cancer patients [61]. ER stress-mediated ATF6 activation resulted in CIP2A upregulation that promoted colon cancer cell survival, whereas CIP2A knockdown exerted cytotoxic effects under ER stress [61]. We have then compared DOX-mediated ER stress with the effects promoted by a well-established inducer of ER stress, namely tunicamycin (TUN) (Fig. 3A).

TUN, an antibiotic and ER stress inducer, is an inhibitor of *N*-linked glycosylation by blocking the transfer of UDP-*N*-acetylglucosamine to dolichol phosphate in the ER [62]. TUN is considered as a promising anticancer agent, because TUN treatment can induce apoptosis and sensitize cancer cells to chemotherapy and radiotherapy [63–67]. As TUN, when used at the concentration of 10 µg/ml for 24 h, promoted a significant increase in the levels of phosphorylated eIF2α in BeWo choriocarcinoma cells [26], we decided to use the same experimental setup to study the effects of *DNMT2/TRDMT1* gene knockout on the activation of eIF2α under TUN-induced UPR and ER stress (Fig. 3A). Indeed, TUN treatment resulted in the activation of eIF2α in four cancer cell lines and *DNMT2/TRDMT1* gene knockout compromised this response (Fig. 3A). TUN also promoted mild and severe cytotoxicity (apoptosis induction, Annexin staining) in MDA-MB-231 and U-2 OS D-NIC cells when cells were treated for 24 and 48 h, respectively (Fig. 3B, C). This was also correlated with elevated caspase activation in U-2 OS D-NIC cells compared to U-2 OS C-NIC cells (Fig. S3). To the best of our knowledge, there is no information on the role of DNMT2/TRDMT1 during ER stress and the UPR. It has been documented that Dnmt2-mediated C38 tRNA methylation is required for the codon fidelity for accurate polypeptide synthesis in a mouse model [24]. The authors speculated that the lack of Dnmt2 may contribute to the accumulation of misfolded proteins and modulate cell fate, but the effect of *Dnmt2* knockout on the UPR signaling was not addressed. Indeed, we have shown that *DNMT2/TRDMT1* gene knockout affected TUN-mediated eIF2α-based UPR signaling and, if TUN treatment was prolonged, a massive apoptotic cell death was induced in cells lacking active *DNMT2/TRDMT1* gene (Fig. 3B, C; Fig. S3). In contrast, *DNMT2/TRDMT1* gene knockout compromised DOX-induced PERK activation (Fig. 2). This effect was also correlated with elevated cytotoxicity when cells were stimulated with 1 µM DOX for 48 h (Fig. S2).

### The effect of *DNMT2/TRDMT1* gene knockout on DOX-mediated stress granules formation and changes in F-actin cytoskeleton

ER stress-induced activation of PERK arm of the UPR, a part of integrated stress response (ISR), resulted in eIF2α phosphorylation-mediated inhibition of global Cap-dependent translation [68]. Indeed, DOX-mediated ER stress was also accompanied by elevated phosphorylated levels of eIF2α in three cancer cell lines (Fig. 2). Stress conditions associated with translational inhibition are also characterized by the formation of cytoplasmic condensates and membrane-less organelles, namely stress granules (SGs) containing mRNA, RNA-binding proteins, translation initiation factors and other components [69]. The biological role of SGs is not fully recognized, but SGs are suggested to be implicated in translational control, mRNA storage, regulation of mRNA decay, antiviral innate immune response and modulation of transduction pathways [69]. As DNMT2/TRDMT1 can be localized in SGs during stress conditions, for example, dithiothreitol treatment, and eIF4E is a DNMT2/TRDMT1-interacting protein [70], the levels of eIF4E and eIF4E-containing SGs were then investigated (Fig. S4). Indeed, increased levels of eIF4E were observed upon 1 µM DOX stimulation in four cancer cell lines (Fig. S4). The effect was more pronounced in C-NIC cells than in D-NIC cells (Fig. S4). Except of U-2 OS cells, 10 times lower concentration of DOX, namely 0.1 µM, also augmented eIF4E levels (Fig. S4). However, eIF4E-containing SGs were predominantly observed when 0.1 µM DOX was used (Fig. S4). At higher concentration, 1 µM DOX, eIF4E signals were more diffused in the cytoplasm (Fig. S4A). Although the authors did not consider to study the components of ER stress signaling, they used DTT as a stressor that is also an inducer of ER stress, thus, the UPR might be initiated while DNMT2/TRDMT1 was localized in SGs [70]. This may suggest that DNMT2/TRDMT1 is involved in SGs-mediated adaptive response during ER stress. As c-Myc activation promoted oxidative stress-mediated increase in DNMT2/TRDMT1 levels and F-actin cytoskeleton remodeling in medulloblastoma cells [71], we decided then to analyze changes in F-actin cytoskeleton after DOX exposure and *DNMT2/TRDMT1* gene knockout (Fig. S4). The effects were cellular context-dependent (Fig. S4B). 1 µM DOX treatment decreased average fiber length in MDA-MB-231 C-NIC and D-NIC cells, whereas 1 µM DOX-mediated increase in average fiber length in U-251 MG C-NIC and D-NIC cells was observed (Fig. S4B). No effect was noticed in DOX-treated U-2 OS cells (Fig. S4B). In general, DOX promoted F-actin heterogeneity in cancer cells (Fig. S4B).

### *DNMT2/TRDMT1* gene knockout affects NSUN-based responses upon DOX treatment

**Fig. 4:**
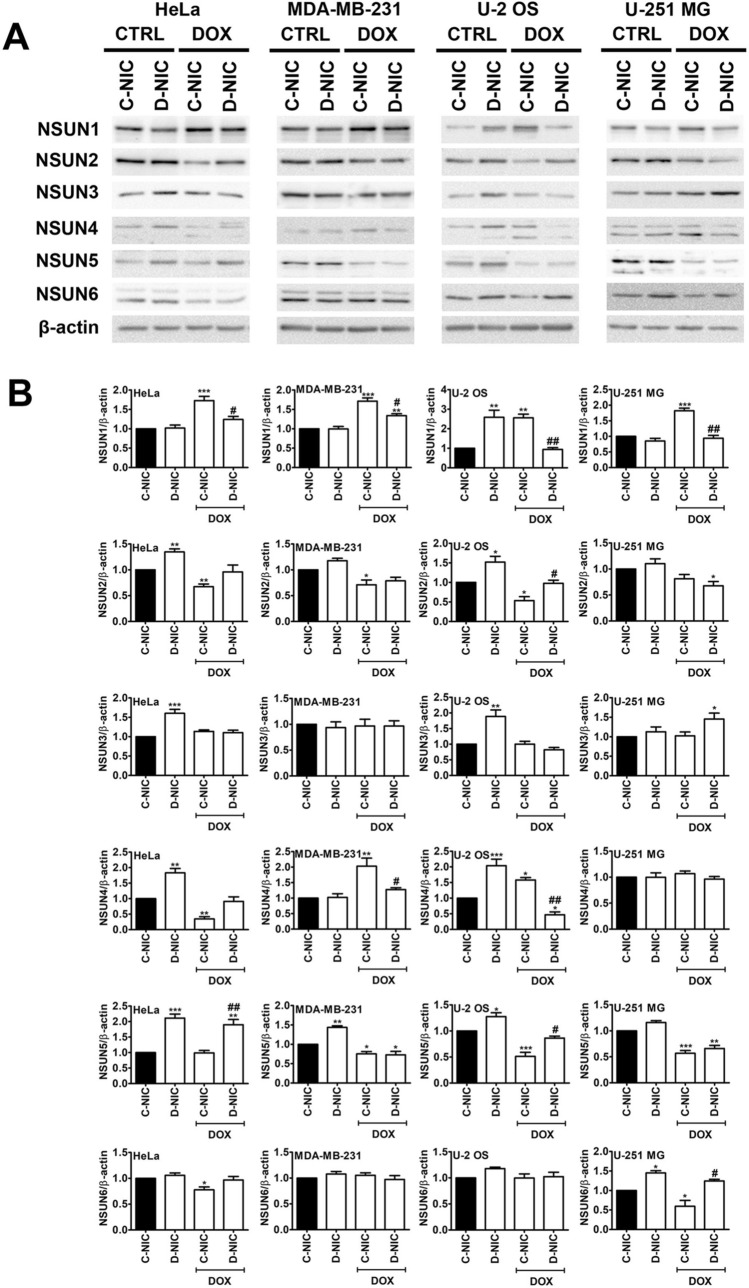
The effect of *DNMT2/TRDMT1* gene knockout on DOX-mediated changes in the levels of NSUN proteins in four cancer cell lines. **A** Western blot-based analysis of the levels of NSUN1, NSUN2, NSUN3, NSUN4, NSUN5 and NSUN6. β-Actin antibody was considered as a loading control. **B** Data were normalized to C-NIC untreated control. Bars indicate SD, n = 3, ***p < 0.001, **p < 0.01, *p < 0.05 compared to untreated C-NIC cells (ANOVA and Dunnett’s a posteriori test), ^##^*p* < 0.01, ^#^*p* < 0.05 compared to drug-treated C-NIC cells (ANOVA and Tukey’s a posteriori test). *CTRL* control conditions, *DOX* doxorubicin treatment, *C-NIC* control cells with unaffected levels of DNMT2/TRDMT1, *D-NIC* cells with *DNMT2/TRDMT1* gene knockout

As the members of the NOL1/NOP2/SUN domain (NSUN) protein family are also m^5^C RNA methyltransferases like DNMT2/TRDMT1 [72], we decided then to evaluate the levels of NSUN1, NSUN2, NSUN3, NSUN4, NSUN5 and NSUN6 in four cancer cell lines lacking active *DNMT2/TRDMT1* gene and upon DOX stimulation (Fig. 4).

*DNMT2/TRDMT1* gene knockout resulted in increased levels of NSUN1, NSUN2, NSUN3, NSUN4 and NSUN5 in U-2 OS D-NIC cells and elevated levels of NSUN3 and NSUN5 in HeLa D-NIC cells in control conditions (Fig. 4). Changes in the levels of NSUNs may be dependent on cell density and culture protocol. Indeed, we have previously observed a different pattern of changes in the levels of NSUNs in *DNMT2/TRDMT1* gene knockout cancer cells, which were cultured for prolonged time at high density [19]. DOX treatment promoted an increase in the levels of NSUN1 in four cancer C-NIC cells (Fig. 4). As this effect was not observed in corresponding D-NIC cells, one can conclude that *DNMT2/TRDMT1* gene knockout compromises NSUN1-based response to DOX exposure (Fig. 4). DOX also stimulated the levels of NSUN4 in MDA-MB-231 and U-2 OS cells C-NIC cells, but not in corresponding D-NIC cells (Fig. 4). In contrast, DOX caused an increase in NSUN3 levels in U-251 MG D-NIC cells, but not in corresponding C-NIC cells (Fig. 4). More recently, we have shown that, except of NSUN2, NSUN protein levels were increased during DOX-induced senescence in four cancer cell lines [19]. The role of NSUN proteins in cancer biology is rather complex as both NSUN-mediated cancer promoting and inhibiting effects were documented [73–77]. For example, NSUN2 stimulated gastric cancer cell proliferation by repressing p57 Kip2 by an m^5^C-dependent manner [75]. In contrast, NSUN6 limited proliferation and development of liver and pancreatic cancer cells [76, 77]. We have previously also observed that *DNMT2/TRDMT1* gene knockout exerted limited effect on the cytoplasmic levels of 5-methylcytosine that may be interpreted as a compensatory effect of increased levels of NSUN proteins in DOX-induced senescent cancer cells [19]. NSUN proteins may also methylate N^6^-adenosine in mammalian microRNAs that result in the formation of N^6^-methyladenosine (m^6^A) [78]. For example, NSUN2-mediated methylation may modulate miR-125b levels and function in response to oxidative stress that resulted in increased levels of p53, Bak1, ErbB2, E2F3 and ppp1ca in hydrogen peroxide-treated HeLa cells [78]. Stress-induced changes in the levels of NSUN proteins may modulate the methylation status of selected microRNAs, thereby repressing microRNA function in gene silencing [78].

### *DNMT2/TRDMT1* gene knockout modulates microRNA profiles in control conditions and upon DOX stimulation in four cancer cell lines

**Fig. 5:**
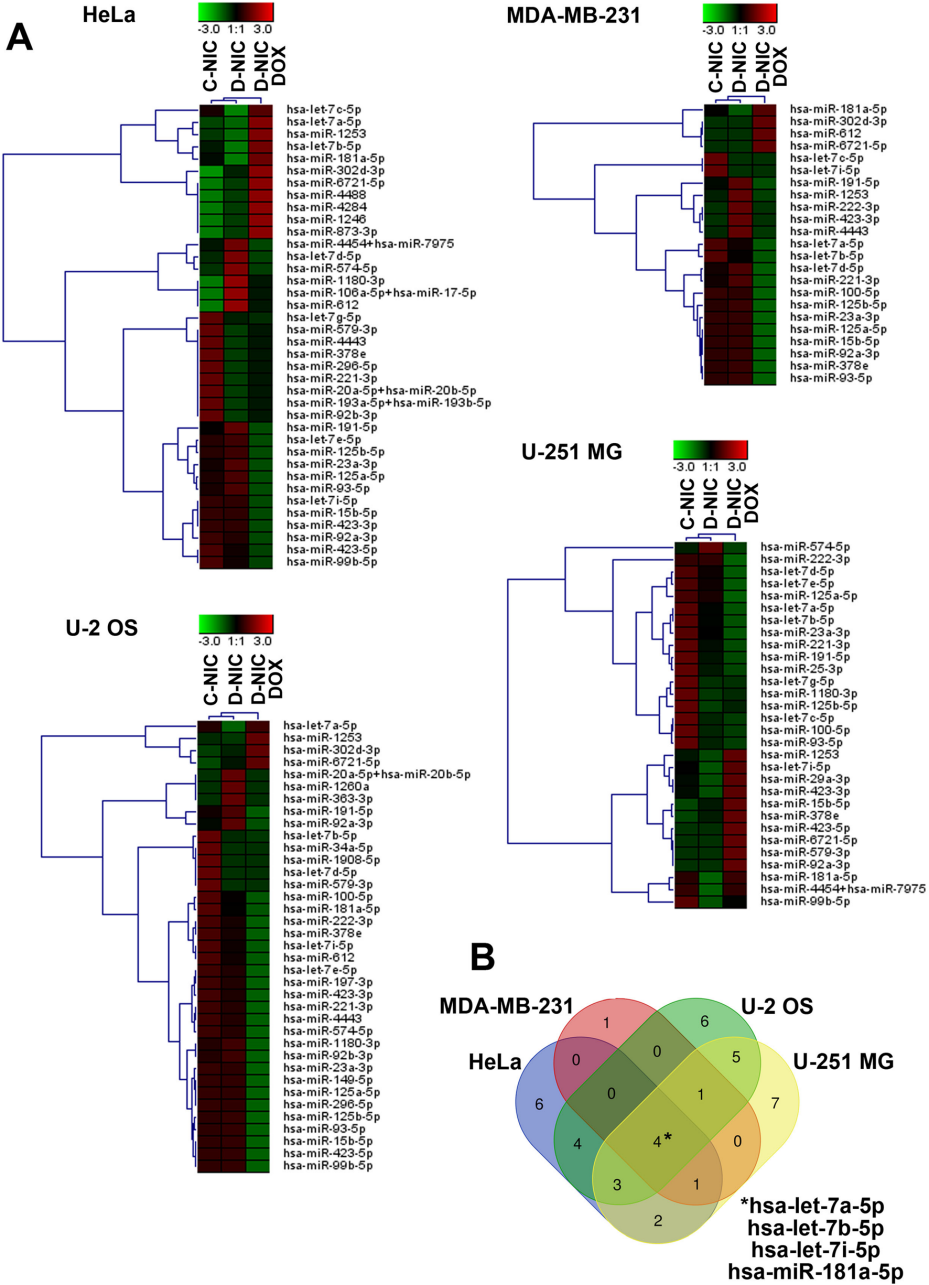
The effect of *DNMT2/TRDMT1* gene knockout on DOX-mediated changes in microRNA profiles in four cancer cell lines. For microRNA profiling, nCounter Human v2 miRNA Panel containing 798 unique microRNA barcodes was used. **A** Heat maps generated from microRNA expression data are shown. Hierarchical clustering was created using Genesis 1.8.1 software. **B** Venn diagram analysis was used to establish the intersection between down-regulated microRNAs in four cancer cell lines with *DNMT2/TRDMT1* gene knockout (D-NIC cells) compared to control cells (C-NIC cells) in control conditions. Four microRNAs with significantly lowered levels (*p* < 0.05) in all four cancer D-NIC cells compared to C-NIC cells were revealed, namely hsa-let-7a-5p, hsa-let-7b-5p, hsa-let-7i-5p and hsa-miR-181a-5p. *DOX* doxorubicin treatment, *C-NIC* control cells with unaffected levels of DNMT2/TRDMT1, *D-NIC* cells with *DNMT2/TRDMT1* gene knockout

We have previously observed that *DNMT2/TRDMT1* silencing affected microRNA pools in human normal fibroblasts [16]. *DNMT2/TRDMT1* silencing resulted in elevated levels of proliferation-related and tumor suppressor microRNAs such as miR-28-3p, miR-34a-3p, miR-30b-5p, miR-29b-3p, miR-200c-3p, miR-28-5p, miR-379-5p, miR-382-5p, miR-194-5p, miR-193b-3p and miR-409-3p [16]. We have suggested that *DNMT2/TRDMT1* may be a regulator of cell proliferation and longevity in human fibroblasts [16]. Thus, it seems interesting to study the effect of *DNMT2/TRDMT1* gene knockout on microRNA profiles also in cancer cells and identify affected genes and pathways. In the present study, the expression of 798 microRNAs was considered in four cancer cell lines lacking active *DNMT2/TRDMT1* gene and upon DOX stimulation using nCounter microRNA expression assay (Fig. 5).

**Fig. 6:**
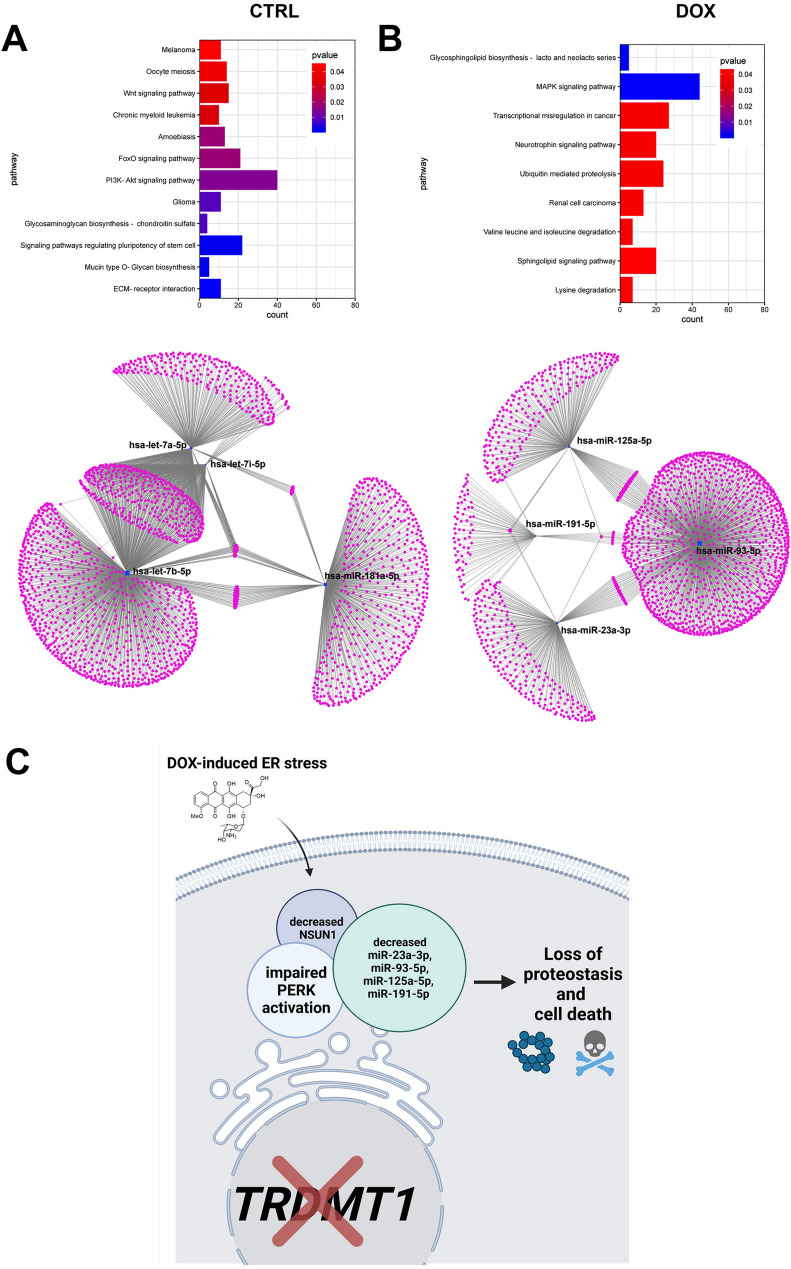
Gene and pathway target analysis for selected microRNA with significantly affected levels in four D-NIC cancer cell lines compared to corresponding C-NIC cells in control conditions (**A** hsa-let-7a-5p, hsa-let-7b-5p, hsa-let-7i-5p and hsa-miR-181a-5p) and upon DOX stimulation (**B** hsa-miR-23a-3p, hsa-miR-93-5p, hsa-miR-125a-5p and hsa-miR-191-5p). Enriched horizontal bars with colors are shown (upper panels). Pathway enrichment analysis was performed using WeiShengxin software. MiRNet and database miRTarBase were used for the visualization of microRNA gene networks (1947 genes for C-NIC cells versus D-NIC cells and 1713 genes for D-NIC cells in control conditions versus DOX-treated D-NIC cells, lower panels). *CTRL* control conditions, *DOX* doxorubicin treatment. **C** A summarizing scheme. *DNMT2/TRDMT1* gene knockout affected PERK-based adaptive response during DOX-induced ER stress as judged by compromised PERK activation in cancer cells. The lack of functional *DNMT2/TRDMT1* gene was accompanied by attenuated NSUN1-mediated response and decreased levels of four microRNAs, namely hsa-miR-23a-3p, hsa-miR-93-5p, hsa-miR-125a-5p and hsa-miR-191-5p involved in the regulation of protein homeostasis and translational control pathways. The loss of *DNMT2/TRDMT1* gene contributes to proteotoxic stress during DOX-induced ER stress that in the case of prolonged ER stress resulted in apoptotic cell death (color figure online)

Hierarchical clustering revealed that untreated C-NIC and D-NIC samples were grouped together in four cancer genetic backgrounds, whereas DOX-treated samples were more different and grouped as a separate category (Fig. 5A). Of course, each cancer cell line responded differently with unique patterns of up- and down-regulated microRNAs (Fig. 5A). However, we would like to establish a common pattern of changes in microRNA expression in four cancer cell lines. Using Venn diagram analysis, decreased levels of four microRNAs, namely hsa-let-7a-5p, hsa-let-7b-5p, hsa-let-7i-5p and hsa-miR-181a-5p were observed in four different cancer cell lines lacking *DNMT2/TRDMT1* gene (Fig. 5B). Similarly, a decrease in the levels of hsa-miR-23a-3p, hsa-miR-93-5p, hsa-miR-125a-5p and hsa-miR-191-5p was noticed in all four D-NIC cells treated with DOX compared to untreated D-NIC cells (data not shown). Changes in the levels of hsa-let-7a-5p, hsa-let-7b-5p, hsa-let-7i-5p, hsa-miR-181a-5p, hsa-miR-23a-3p, hsa-miR-93-5p, hsa-miR-125a-5p and hsa-miR-191-5p upon DOX stimulation and *DNMT2/TRDMT1* gene knockout were also validated using a real-time PCR approach (Fig. S5). Indeed, a similar pattern of statistically significant decrease in the levels of selected microRNAs was observed (Fig. S5). We have then considered a pathway enrichment analysis to reveal the most significantly affected pathways by selected microRNAs (Fig. 6A, B).

1947 Genes were identified to be modulated by hsa-let-7a-5p, hsa-let-7b-5p, hsa-let-7i-5p and hsa-miR-181a-5p (gene regulatory network 1, Fig. 6A) and 1713 genes were revealed to be affected by hsa-miR-23a-3p, hsa-miR-93-5p, hsa-miR-125a-5p and hsa-miR-191-5p (gene regulatory network 2, Fig. 6B). For gene regulatory network 1, several pathways were documented, for example, PI3K-AKT signaling pathway, FoxO signaling pathway and signaling pathways regulating pluripotency of stem cell (Fig. 6A). We have also identified genes that were regulated simultaneously by four microRNAs (hsa-let-7a-5p, hsa-let-7b-5p, hsa-let-7i-5p and hsa-miR-181a-5p) with decreased levels in four cancer cell lines lacking *DNMT2/TRDMT1* gene (Fig. 6A), namely *ZC3HAV1L*, *NHLRC3*, *NR6A1*, *HMGA2*, *AHR*, *TUBB2A*, *SLC10A7*, *ZNF556*, *SMCR8*, *NCOA3*, *HUWE1*, *CCNG1*, *MTUS1*, *YOD1*, *PHACTR4*, *CDKN1A*, *FAM222B*, *PMAIP1*, *TMED4*, *MTX3*, *TGFBR3*, *ND2*, *HRAS*, *TAB2*, *BAZ2A* and *NRAS*. These genes are encoding, for example, cell cycle regulators, cytoskeletal proteins, transcription factors and proto-oncogenes. In contrast, for gene regulatory network 2, the most significantly enriched pathways were MAPK signaling pathway, transcriptional misregulation in cancer, ubiquitin-mediated proteolysis and others (Fig. 6B). We were unable to reveal a group of genes that would be simultaneously regulated by four microRNAs (hsa-miR-23a-3p, hsa-miR-93-5p, hsa-miR-125a-5p and hsa-miR-191-5p) with decreased levels in DOX-treated four cancer cell lines lacking *DNMT2/TRDMT1* gene (Fig. 6B).

The relationships between ER stress and microRNAs are rather complex as they can regulate each other and collaborate to determine cancer cell fate [79–82]. A number of ER stress components can be a target of selected microRNAs and microRNAs can be regulated by ER stress sensor-mediated microRNA biogenesis [79–82]. For example, under ER stress, IRE1α can cleave selected microRNAs, namely miR-17, miR-34a, miR-96 and miR-125b, to derepress translation of proapoptotic caspase-2 [83]. Under ER stress, oncogenic microRNAs (e.g., miR-30b-5p, mir-30c-5p, miR-451a) can be also upregulated and tumor suppressor microRNAs (e.g., miR-183, miR-1992, miR-214) can be downregulated promoting cancer cell survival and inhibiting cancer cell death [82]. *DNMT2/TRDMT1* gene knockout in four different genetic backgrounds also promoted a decrease in the levels of four microRNAs, namely miR-23a-3p, miR-93-5p, miR-125a-5p and miR-191-5p during DOX-induced ER stress (this study). Except of miR-23a, there is no information about the involvement of miR-93-5p, miR-125a-5p and miR-191-5p in the regulation of ER stress signaling. In HeLa cells, ER stress-mediated miR-23a downregulation resulted in elevated expression of lactate dehydrogenase (LDH) that promoted aerobic glycolysis-based pro-survival response [84]. ER stress-associated release of exosomal miR-23a-3p from hepatoma cells also promoted immune escape by miR-23a-PTEN-AKT pathway-mediated PD-L1 upregulation and T-cell function inhibition [85].

In conclusion, we have documented that the lack of active *DNMT2/TRDMT1* gene compromised PERK-mediated response to DOX-induced ER stress in different cancer cells that was accompanied by changes in the levels of NSUN proteins and a decrease in the pools of four microRNAs, namely miR-23a-3p, miR-93-5p, miR-125a-5p and miR-191-5p with regulatory roles in maintaining proteostasis and controlling translation (Fig. 6C). Furthermore, when DOX treatment was prolonged, the accumulation of protein aggregates and apoptotic cells death were documented in cells lacking active *DNMT2/TRDMT1* gene (Fig. 1E; Fig. S2). *DNMT2/TRDMT1* gene knockout also affected TUN-mediated eIF2α-based UPR signaling that resulted in increased cytotoxicity when ER stress was prolonged (Fig. 3; Fig. S3). As the importance of DNMT2/TRDMT1 function(s) during a plethora of cellular stress responses was reported in normal and cancer cells [12–20, 22, 70] and DNMT2/TRDMT1 contributed to translational fidelity and accurate protein synthesis [24], and modulated the UPR (this study), DNMT2/TRDMT1 may be suggested to be a novel target in ER stress-based anticancer therapy.

## Supplementary Information

Below is the link to the electronic supplementary material.

Supplementary material 1 (DOCX 6659 KB)

## Data Availability

The data that support the findings of this study are available from the corresponding author upon reasonable request.
