# Supplementary material for: DNMT2/TRDMT1 gene knockout compromises doxorubicin-induced unfolded protein response and sensitizes cancer cells to ER stress-induced apoptosis
Source: Apoptosis. 2022 Oct 23;28(1-2):166–85. doi: 10.1007/s10495-022-01779-0 (PMC9950192; doi:10.1007/s10495-022-01779-0)
Supplement: Supplementary file 1 — Supplementary material 1 (DOCX 6659 KB) [file 10495_2022_1779_MOESM1_ESM.docx]

**Supplementary information**

**Supplementary Figure 1**

**
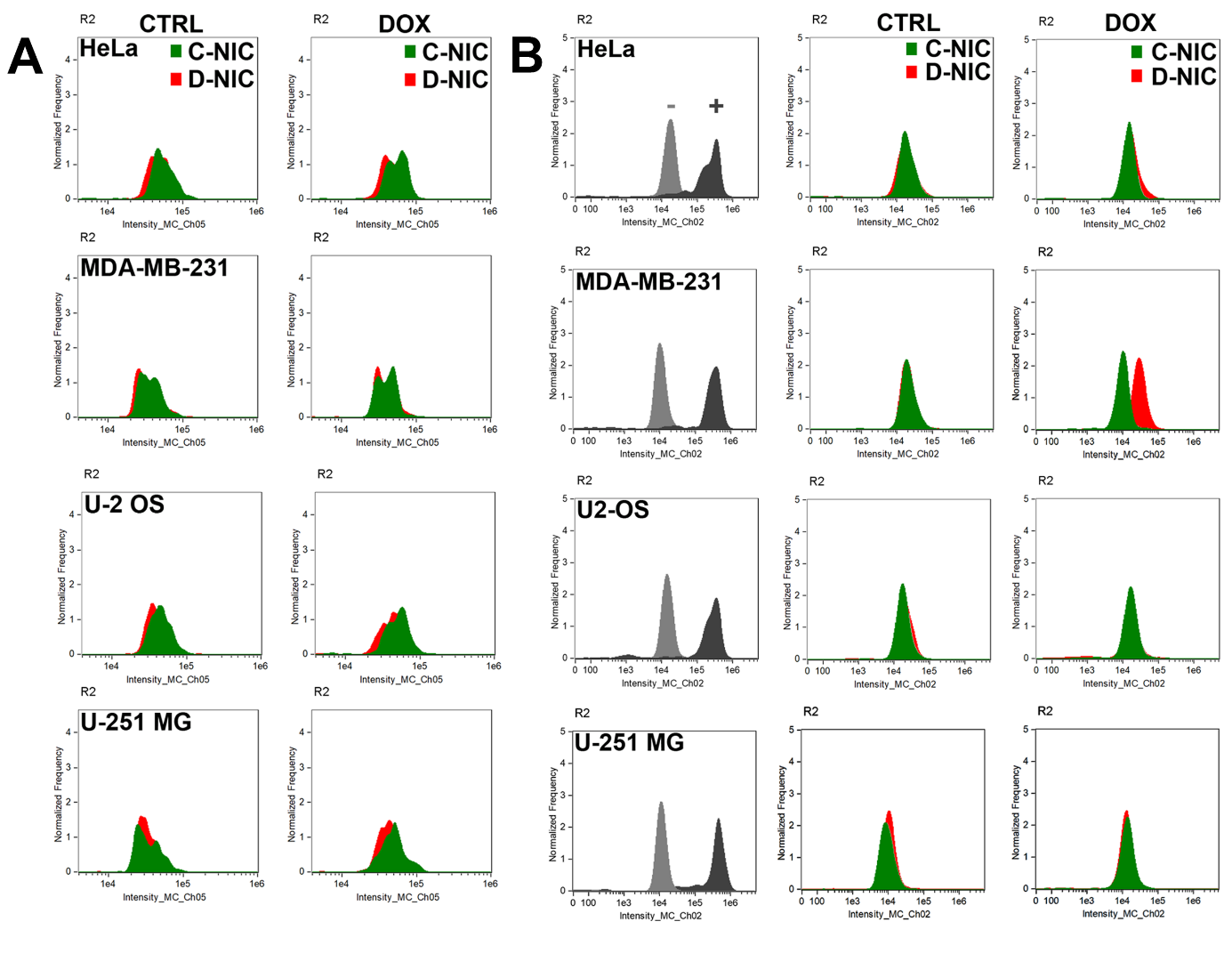
**

**Supplementary Figure 1.** Doxorubicin (DOX)-mediated changes in cell proliferation (A) and DNA fragmentation-mediated apoptosis (B) in four cancer cell lines (HeLa cervical cancer cells, MDA-MB-231 breast cancer cells, U-2 OS osteosarcoma cells and U-251 MG glioblastoma cells) lacking active *DNMT2/TRDMT1* gene (D-NIC cells). Cells were treated with 1 µM DOX for 24 h. (A) Cell proliferation was assayed using Ki67 immunostaining. Ki67 signals were analyzed using imaging flow cytometry. Representative histograms are presented. (B) Imaging flow cytometry-based analysis of DNA fragmentation (TUNEL assay). Representative histograms are presented. Positive controls are also shown (+, dark gray histograms, C-NIC cells treated with DNase). CTRL, control conditions; DOX, doxorubicin treatment; C-NIC, control cells with unaffected levels of DNMT2/TRDMT1; D-NIC, cells with *DNMT2/TRDMT1* gene knockout.

**Supplementary Figure 2A**

**
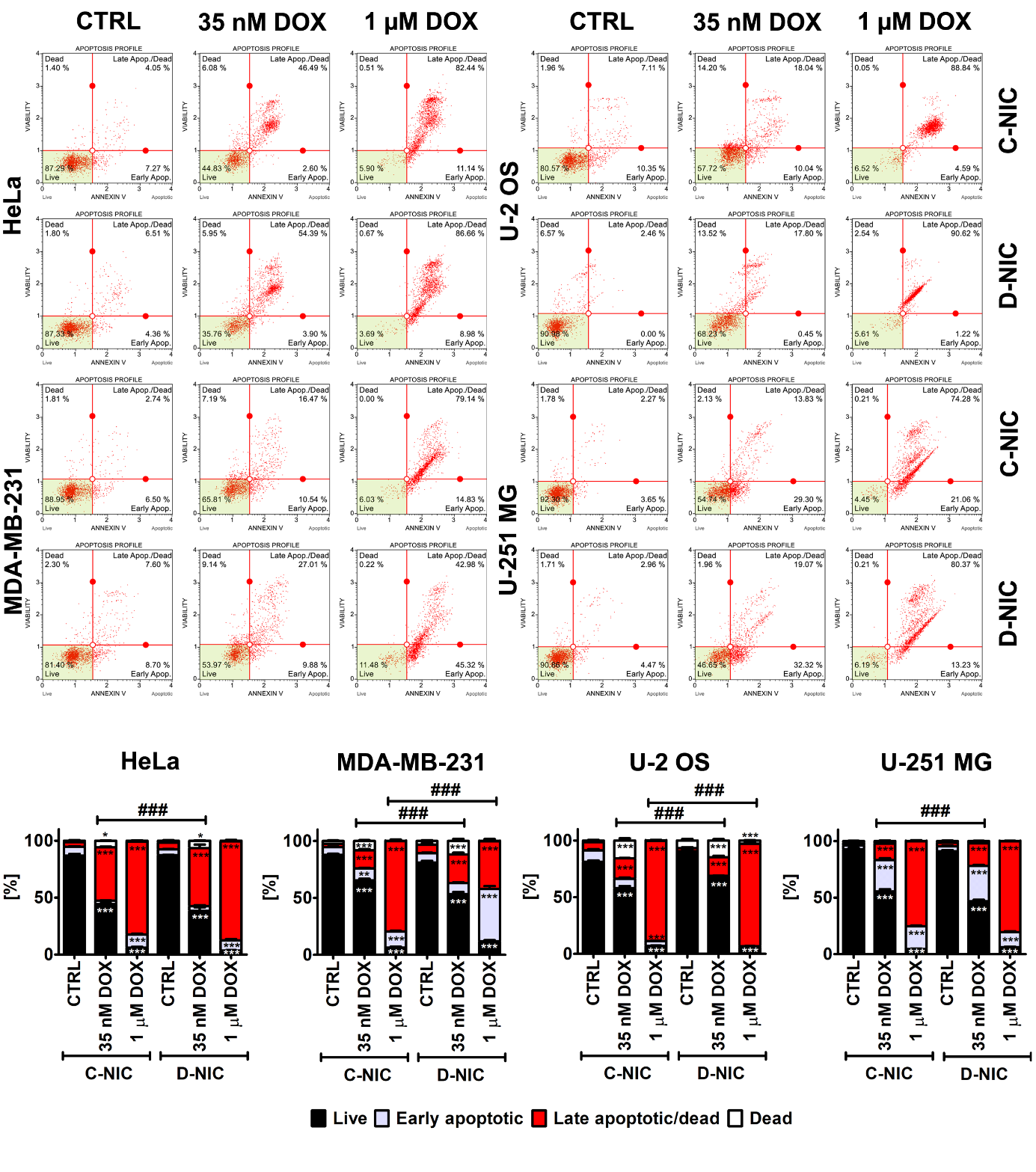
**

**Supplementary Figure 2B**

**
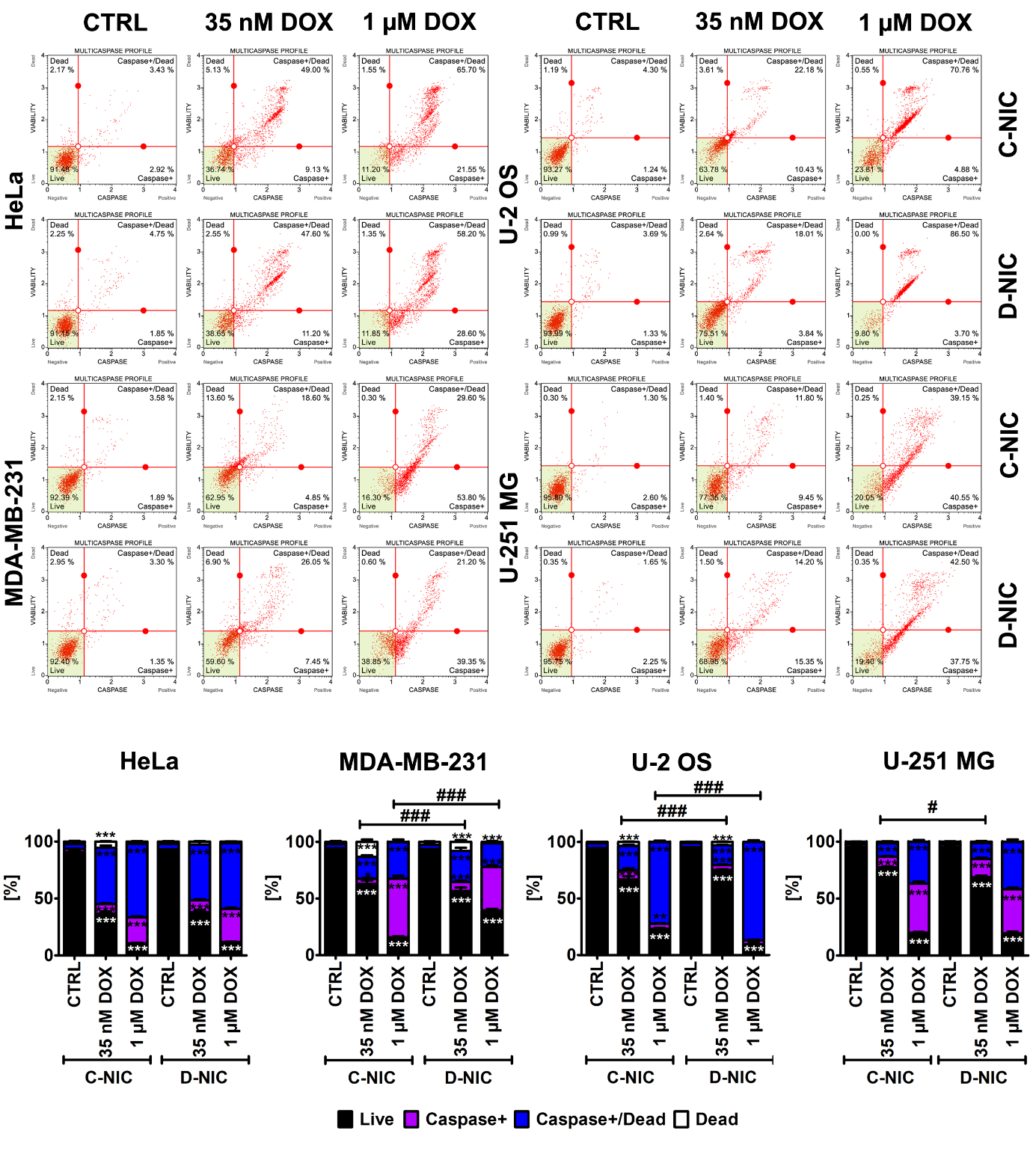
**

**Supplementary Figure 2.** Doxorubicin (DOX)-mediated apoptosis in four cancer cell lines (HeLa cervical cancer cells, MDA-MB-231 breast cancer cells, U-2 OS osteosarcoma cells and U-251 MG glioblastoma cells) lacking active *DNMT2/TRDMT1* gene (D-NIC cells). Cells were treated with 35 nM or 1 µM DOX for 48 h and then two biomarkers of apoptotic cell death were inspected, namely phosphatidylserine externalization (A) and pan-caspase activity (B). Flow cytometry and dedicated reagents and protocols were used. Representative dot plots are shown. Bars indicate SD, n = 3, ^***^*p* < 0.001, ^**^*p* < 0.01, ^*^*p* < 0.05 compared to untreated C-NIC cells (ANOVA and Dunnett’s *a posteriori* test), ^###^*p* < 0.001, ^#^*p* < 0.05 compared to drug-treated C-NIC cells (ANOVA and Tukey’s *a posteriori* test). CTRL, control conditions; DOX, doxorubicin treatment; C-NIC, control cells with unaffected levels of DNMT2/TRDMT1; D-NIC, cells with *DNMT2/TRDMT1* gene knockout.

**Supplementary Figure 3A**

**
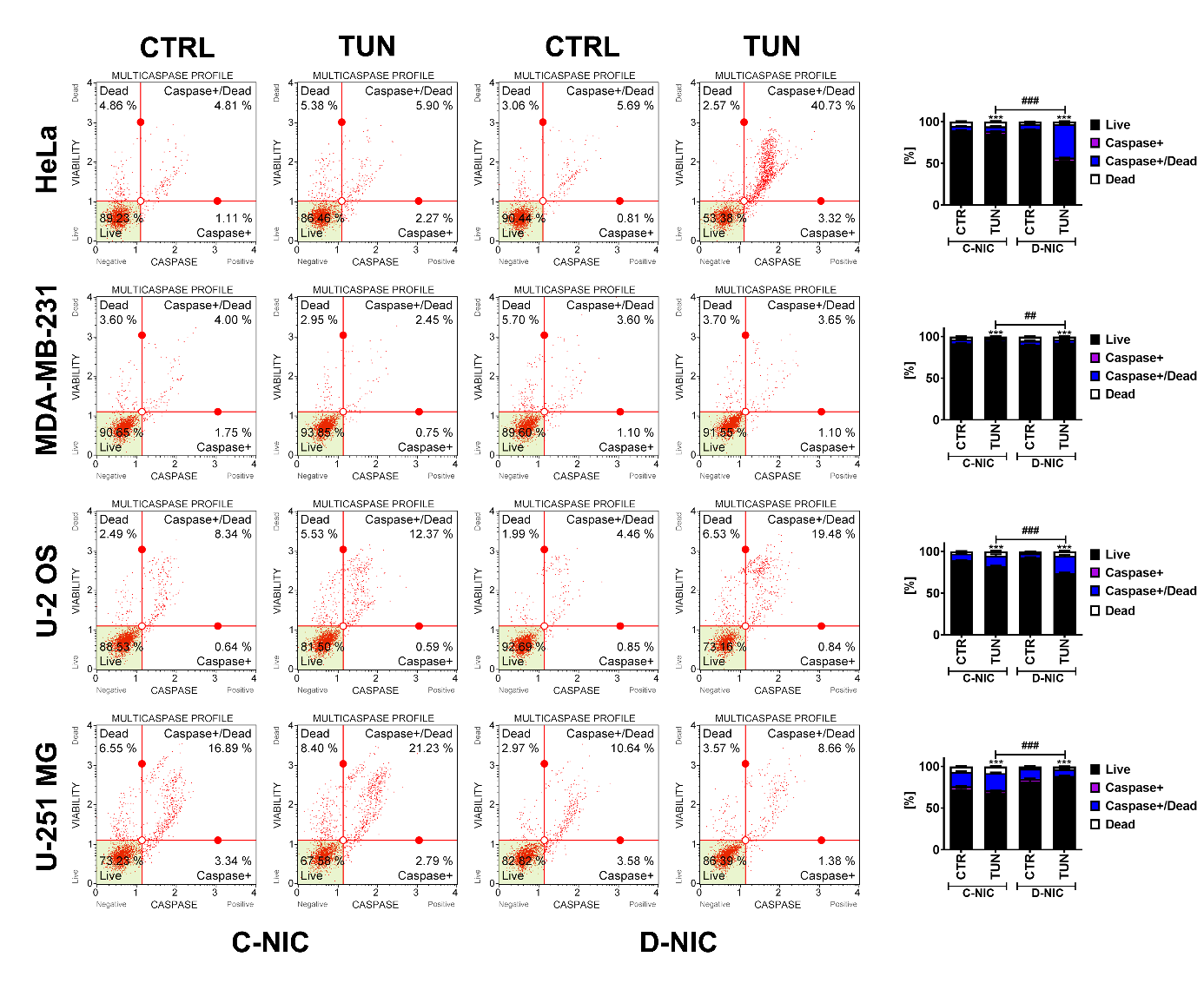
**

**Supplementary Figure 3B**

**
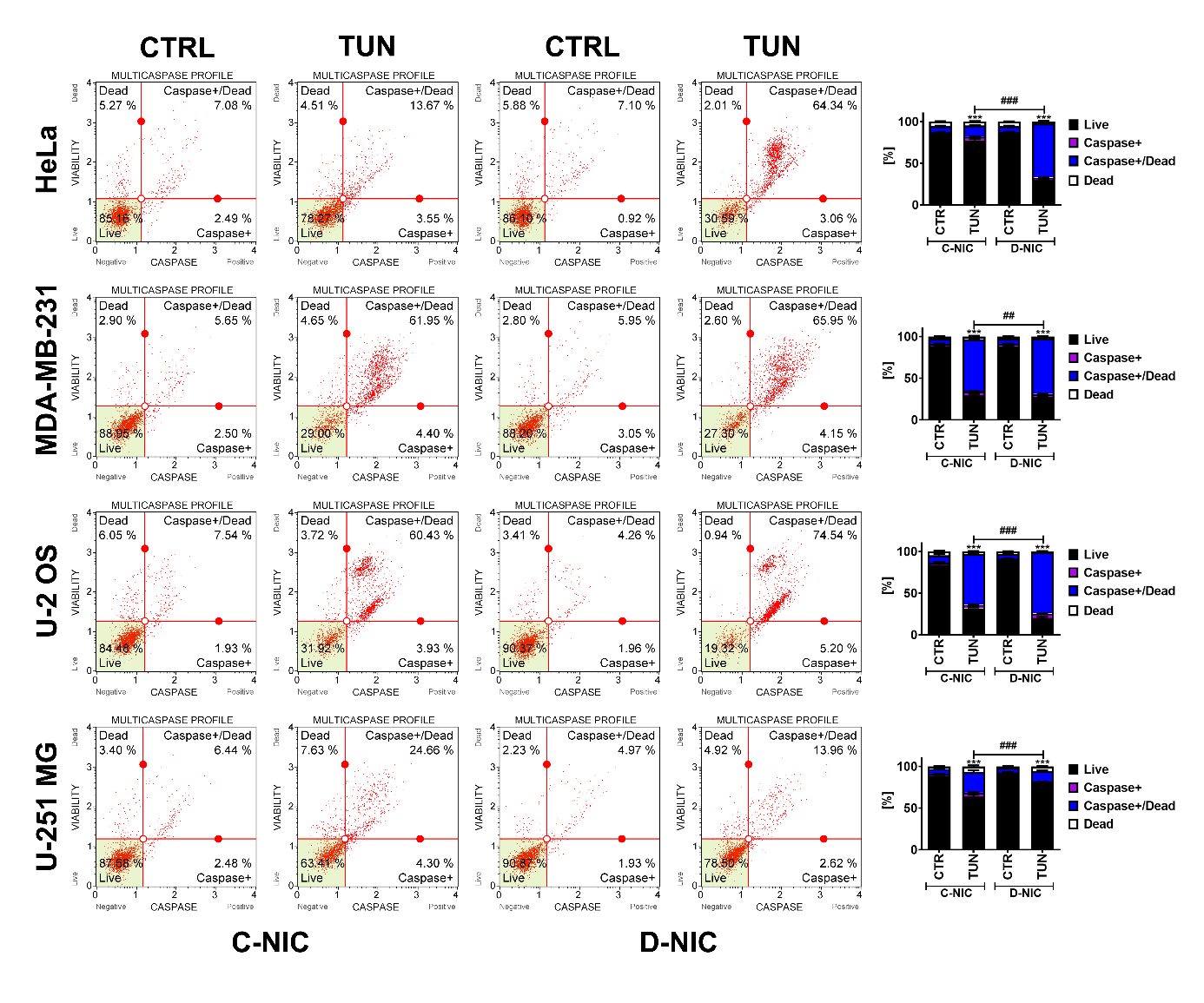
**

**Supplementary Figure 3.** The effect of *DNMT2/TRDMT1* gene knockout on TUN-induced apoptosis (A, B) in four cancer cell lines. After 24 h (A) and 48 h (B) treatment with 10 µg/ml TUN, apoptosis induction was evaluated using flow cytometry and pan-caspase activity test. Representative dot plots are presented. Bars indicate SD, n = 3, ^***^*p* < 0.001 compared to untreated C-NIC cells (ANOVA and Dunnett’s *a posteriori* test), ^###^*p* < 0.001, ^##^*p* < 0.01 compared to drug-treated C-NIC cells (ANOVA and Tukey’s *a posteriori* test). CTRL or CTR, control conditions; TUN, tunicamycin treatment; C-NIC, control cells with unaffected levels of DNMT2/TRDMT1; D-NIC, cells with *DNMT2/TRDMT1* gene knockout.

**Supplementary Figure 4**

**
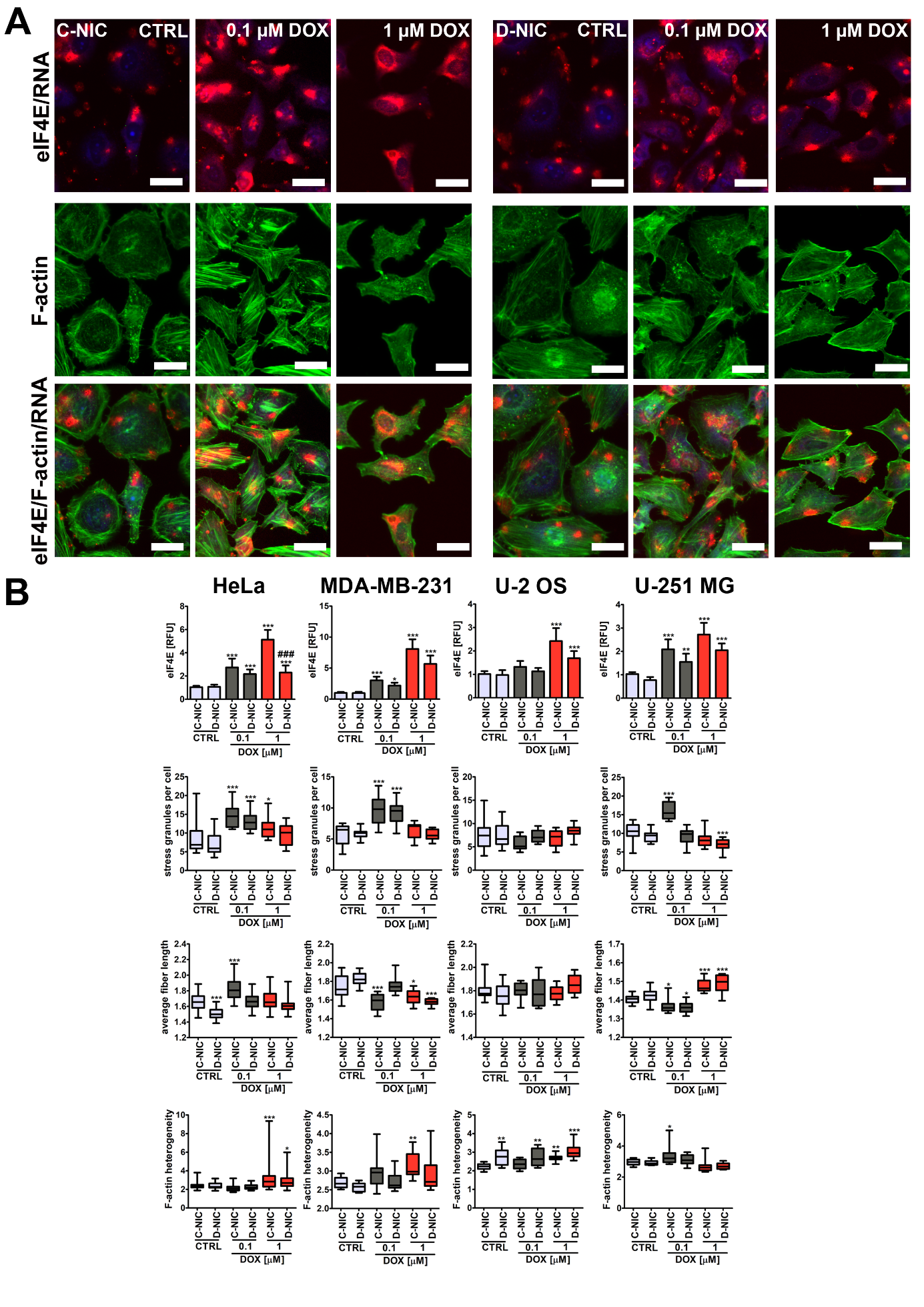
**

**Supplementary Figure 4.** The effect of *DNMT2/TRDMT1* gene knockout on DOX-mediated changes in the levels of eIF4E, formation of stress granules and F-actin cytoskeleton in four cancer cell lines. (A) Monochromatic digital cell images were considered and presented using RGB color system. eiF4E (red), F-actin cytoskeleton (green) and RNA (blue) are presented. Representative microphotographs are shown (HeLa cells), objective 20x. (B) Quantitative analysis of the levels of eIF4E, stress granules, actin fiber length and heterogeneity was conducted using imaging cytometry and dedicated software. eIF4E levels are presented as relative fluorescence units (RFU). Stress granules (puncta with eIF4E immunosignals) were calculated per cell. F-actin cytoskeleton was analyzed using two parameters, namely average fiber length and F-actin heterogeneity. Bars indicate SD or box and whisker plots are shown, n = 3, ^***^*p* < 0.001, ^**^*p* < 0.01, ^*^*p* < 0.05 compared to untreated C-NIC cells (ANOVA and Dunnett’s a posteriori test), ^###^*p* < 0.001 compared to drug-treated C-NIC cells (ANOVA and Tukey’s a posteriori test). CTRL, control conditions; DOX, doxorubicin treatment; C-NIC, control cells with unaffected levels of DNMT2/TRDMT1; D-NIC, cells with *DNMT2/TRDMT1* gene knockout.

**Supplementary Figure 5: Panel I**

**
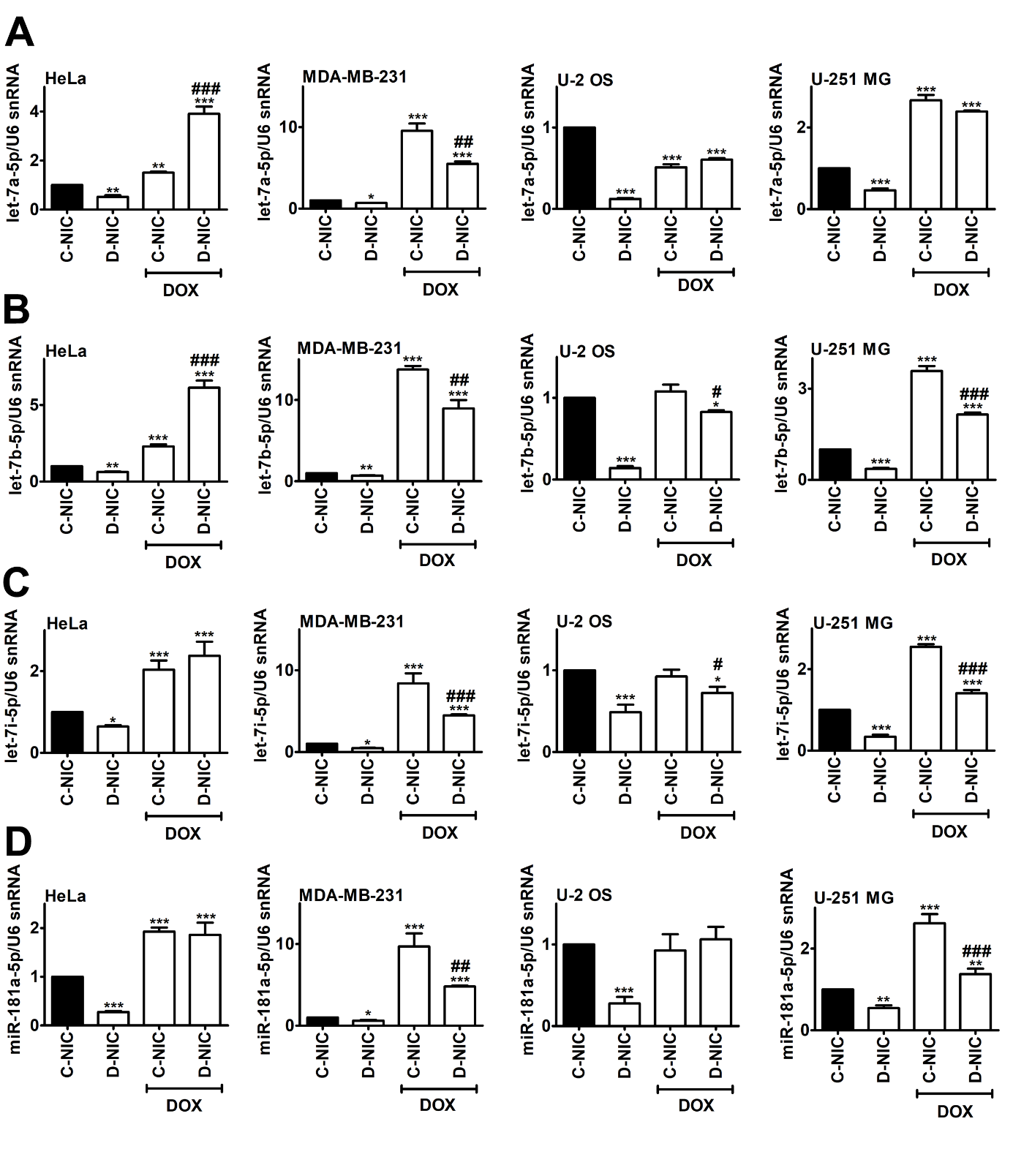
**

**Supplementary Figure 5: Panel II**

**
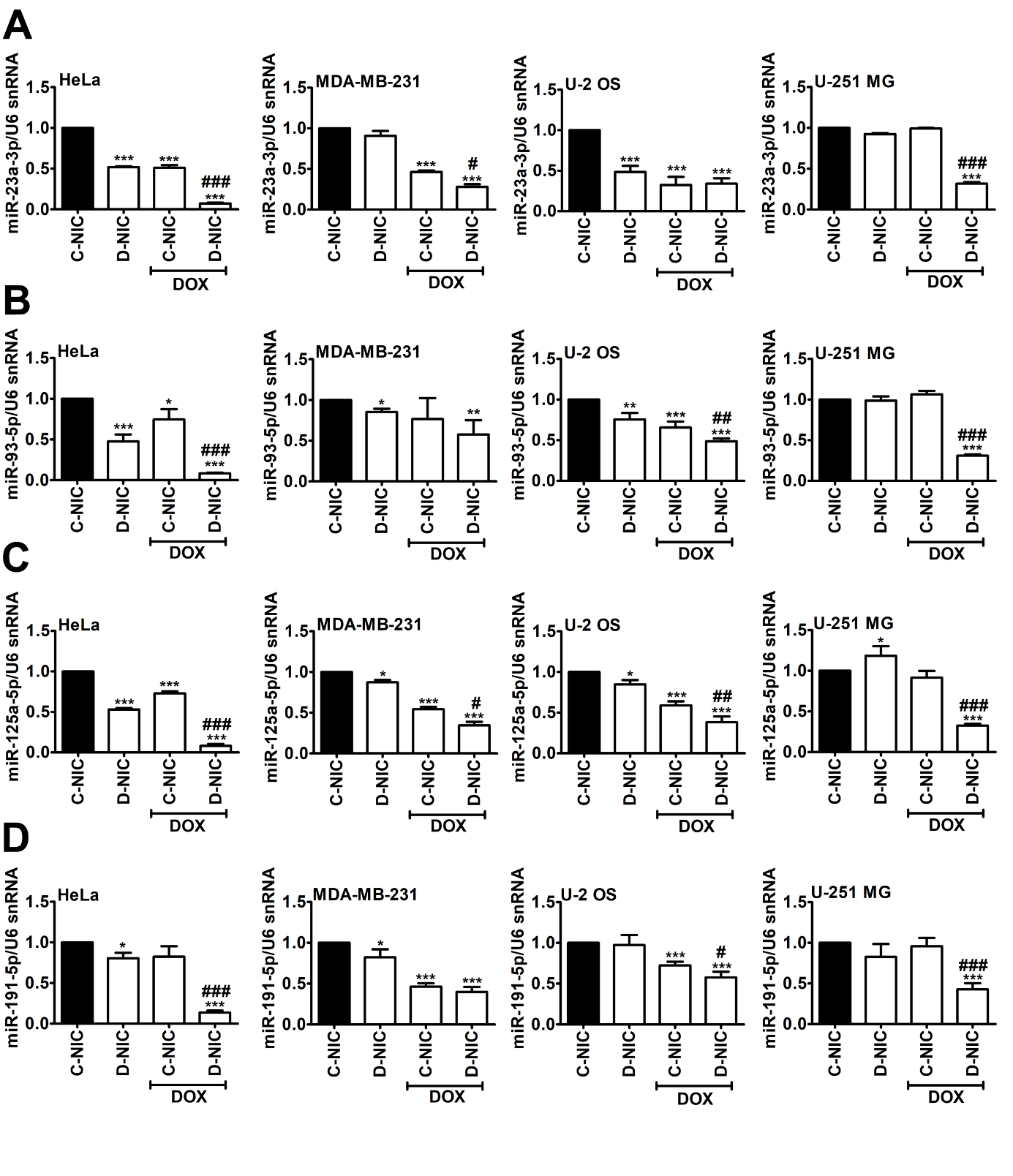
**

**Supplementary Figure 5.** RT-PCR-based validation of observed changes in the levels of selected microRNAs in four cancer cell lines lacking active *DNMT2/TRDMT1* gene (D-NIC cells) using NanoString approach. miRCURY^®^ LNA^®^ miRNA PCR manual, miRCURY LNA RT kit and miRCURY SYBR Green PCR Kit with ROX dye and miRCURY LNA primers (QIAGEN) were used for detection of selected miRNA, namely: hsa-let-7a-5p, hsa-let-7b-5p, hsa-let-7i-5p, hsa-miR-181a-5p (Panel I, A, B, C and D, respectively) and hsa-miR-23a-3p, hsa-miR-93-5p, hsa-miR-125a-5p and hsa-miR-191-5p (Panel II, A, B, C and D, respectively). The U6 snRNA was considered as a reference gene. The results were quantified and compared to corresponding cells with active *DNMT2/TRDMT1* gene (C-NIC) under control conditions. Bars indicate SD, n = 3, ^***^*p* < 0.001, ^**^*p* < 0.01, ^*^*p* < 0.05 compared to untreated C-NIC cells (ANOVA and Dunnett’s *a posteriori* test), ^###^*p* < 0.001, ^##^*p* < 0.01, ^#^*p* < 0.05 compared to drug-treated C-NIC cells (ANOVA and Tukey’s *a posteriori* test). CTRL, control conditions; DOX, doxorubicin treatment; C-NIC, control cells with unaffected levels of DNMT2/TRDMT1; D-NIC, cells with *DNMT2/TRDMT1* gene knockout.
